# Diamond-inclusion system recording old deep lithosphere conditions at Udachnaya (Siberia)

**DOI:** 10.1038/s41598-019-48778-x

**Published:** 2019-08-29

**Authors:** Fabrizio Nestola, Gabriele Zaffiro, Mattia L. Mazzucchelli, Paolo Nimis, Giovanni B. Andreozzi, Benedetta Periotto, Francesco Princivalle, Davide Lenaz, Luciano Secco, Leonardo Pasqualetto, Alla M. Logvinova, Nikolay V. Sobolev, Alessandra Lorenzetti, Jeffrey W. Harris

**Affiliations:** 1 Dipartimento di Geoscienze, Università degli Studi di Padova, Via Gradenigo 6, I-35131 Padova, Italy; 2 Dipartimento di Scienze della Terra e dell’Ambiente, Università degli Studi di Pavia, Via Ferrata 1, I-27100 Pavia, Italy; 3 Dipartimento di Scienze della Terra, Sapienza Università di Roma, Piazzale Aldo Moro 5, I-00185 Roma, Italy; 4 Dipartimento di Matematica e Geoscienze, Università degli Studi di Trieste, Via Weiss 8, I-34127 Trieste, Italy; 5 Institute of Geology and Mineralogy, Russian Academy of Sciences Siberian Branch, Novosibirsk, 630090 Russia; 6 Department of Geology and Geophysics, Novosibirsk State University, Pirogova 2, 630090 Novosibirsk, Russia; 7 Dipartimento di Ingegneria Industriale, Università degli Studi di Padova, Via Marzolo 9, I-35131 Padova, Italy; 8 School of Geographical and Earth Sciences, University of Glasgow, Glasgow, G12 8QQ UK

**Keywords:** Mineralogy, Petrology

## Abstract

Diamonds and their inclusions are unique fragments of deep Earth, which provide rare samples from inaccessible portions of our planet. Inclusion-free diamonds cannot provide information on depth of formation, which could be crucial to understand how the carbon cycle operated in the past. Inclusions in diamonds, which remain uncorrupted over geological times, may instead provide direct records of deep Earth’s evolution. Here, we applied elastic geothermobarometry to a diamond-magnesiochromite (mchr) host-inclusion pair from the Udachnaya kimberlite (Siberia, Russia), one of the most important sources of natural diamonds. By combining X-ray diffraction and Fourier-transform infrared spectroscopy data with a new elastic model, we obtained entrapment conditions, *P*_trap_ = 6.5(2) GPa and *T*_trap_ = 1125(32)–1140(33) °C, for the mchr inclusion. These conditions fall on a ca. 35 mW/m^2^ geotherm and are colder than the great majority of mantle xenoliths from similar depth in the same kimberlite. Our results indicate that cold cratonic conditions persisted for billions of years to at least 200 km in the local lithosphere. The composition of the mchr also indicates that at this depth the lithosphere was, at least locally, ultra-depleted at the time of diamond formation, as opposed to the melt-metasomatized, enriched composition of most xenoliths.

## Introduction

### Cr-spinels in Diamonds

Lithospheric diamonds (those diamonds formed at depths between about 130 and 200 km) represent 99% of all mined diamonds^1^. These diamonds may contain mineral inclusions, which can be used to derive inferences on the physical-chemical environments in which their host diamonds were formed. Cr-rich spinel (mainly magnesiochromite, hereafter, mchr) is one of the most common inclusions found in diamonds, representing about 14% of reported inclusions in lithospheric diamonds worldwide^1^. The compositional variations in mchr inclusions are mainly reflected in their Cr/(Cr + Al) and Mg/(Mg + Fe) molar ratios (hereafter, Cr# and Mg#, respectively). Typical ranges are Cr# = 0.80–0.93 and Mg# = 0.59–0.80, with an average crystal chemical formula of Mg_0.70_Fe_0.36_Cr_1.67_Al_0.26_O_4_^1^. The composition of mchr in mantle peridotites from the diamond stability field depends on pressure (*P*), temperature (*T*), host-rock composition and modal abundance of mchr in the rock^2^. Since the composition of mchr is not a simple function of *P* and *T*, mchr inclusions are not typically used for geothermobarometry. In this work, we adopted the elastic geothermobarometry approach to determine the formation *P* of mchr-bearing diamonds. Given the relatively common occurrence of mchr inclusions in diamonds, this method can potentially be applied to a large number of stones from worldwide localities. This would in turn allow better insight into the depth distribution of lithospheric diamonds at global scale and, consequently, into the processes that control diamond formation in the Earth’s mantle.

### Unprecedented Application of Elastic Geothermobarometry to Diamond-mchr host-Inclusion System

The elastic geothermobarometry approach is based on an old idea^3,4^; in the last few years, its theoretical basis has been extended with the aim to calculate the depth of formation of a diamond-inclusion pair, by combining non-linear equations of state (EoS) with suitable models for elastic relaxation and without any approximations of linear elasticity^5–17^. When a mineral trapped in a host at conditions *P*_*trap*_ and *T*_*trap*_ is exhumed to the Earth’s surface, it may develop residual strains as a result of the contrast in elastic properties between the host and the inclusion. If these residual strains are measured when the host is at room pressure, and *T*_*trap*_ is known independently, *P*_*trap*_ can be back-calculated as the conditions at which there are no strain and stress gradients in the host-inclusion system. This calculation is possible if the elastic properties (*i.e*. bulk modulus, its pressure derivative and the thermal expansion behavior expressed as EoS) of both the host and the inclusion are known. So far, only the EoS for diamond and for a few common mineral inclusions in diamond were available with the accuracy required for geobarometric calculations^7,12,13,18^. In particular, the elastic properties are not only dependent on the mineral phase, but also on its chemical composition. Therefore, to extend the applicability of elastic geobarometry to various inclusion types, it is crucial to determine the EoS for a larger number of minerals and compositions. Moreover, elastic geobarometry could lead to incorrect estimates of entrapment conditions if the following factors are not adequately considered: (i) the occurrence of brittle or plastic deformation in the host diamond; (ii) the shape of the inclusion and its distance from other inclusions and from the external surfaces of the diamond^16^; (iii) the elastic anisotropy of the host and of the inclusion^15,17,19–21^; (iv) the presence of a fluid rim around the inclusions observed in several diamonds^17,22,23^. In this light, mchr inclusions in lithospheric diamonds could be among the best candidates for elastic geobarometry, because (a) plastic deformation is generally minor in lithospheric diamonds compared with higher-T and highly strained sub-lithospheric diamonds; (b) the mchr crystal shape is often equant to almost rounded, thus the “geometrical effect” is small^16^, and (b) both mchr and diamond have a cubic symmetry and their elastic behavior can be approximated as isotropic. A possible problem is the presence of a fluid rim, which is quite common at the interface between mchr and diamond^22^. The elastic properties of such fluid are not known, but its estimated thickness is so limited (even less than 1 μm) that its effect can reasonably be neglected, at least in the absence of cracks departing from the host–inclusion interface.

**Figure 1:**
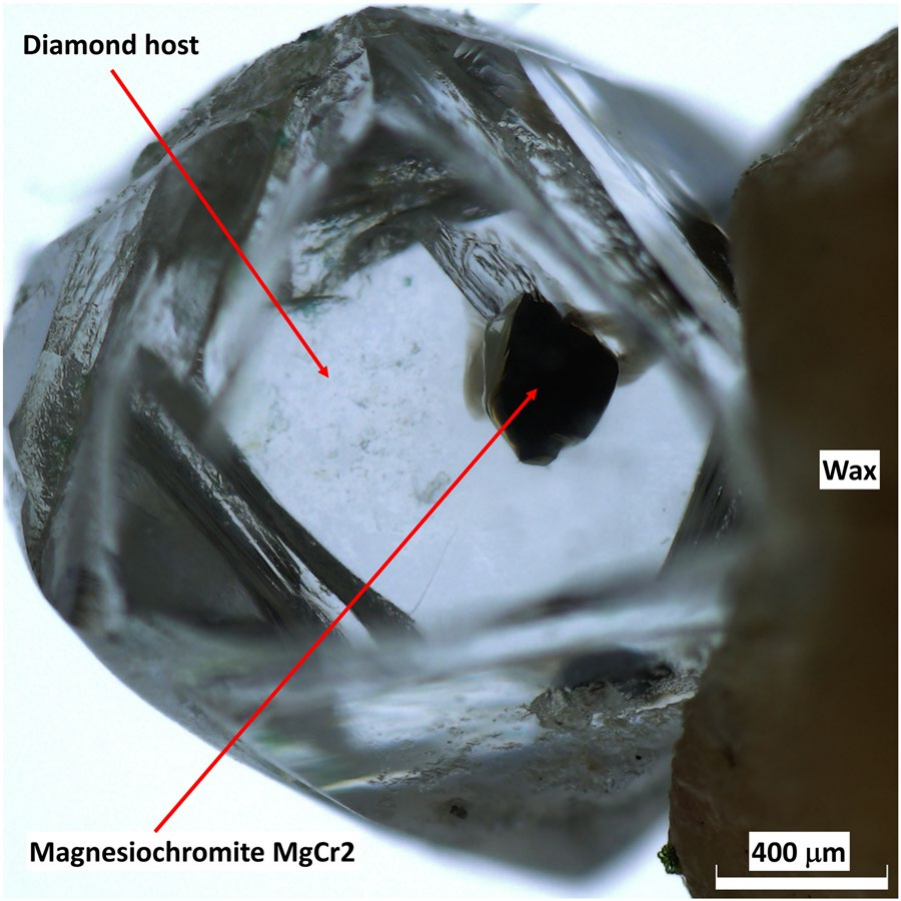
MgCr2 inclusion still trapped within its diamond host, mounted on wax for SC-XRD measurements.

At present, the main obstacle to the application of elastic geobarometry to mchr-bearing diamonds is the knowledge of the EoS of mchr. The variations in published values for bulk modulus and its first pressure derivative for mchr with different compositions^24–26^ indicate a degree of uncertainty that is too large for reliable geobarometry. To circumvent this problem, we have determined experimentally an EoS for a single mchr crystal, sample Ac139, which had been extracted from a Siberian diamond. We used state-of-art high-pressure techniques (see Methods) in order to get accurate and precise unit-cell volumes as a function of *P*. The resulting EoS was applied to a second mchr inclusion, sample MgCr2, Fig. 1, in another Siberian diamond from the Udachnaya kimberlite with the aim of determining the depth of diamond formation.

The chemical compositions of Ac139 and MgCr2 are the following:

**Ac139**: Mg_0.64_Fe_0.40_Cr_1.73_Al_0.22_Ti_0.01_Mn_0.01_O_4_

**MgCr2**: Mg_0.64_Fe_0.42_Cr_1.66_Al_0.26_Ti_0.02_V_0.04_O_4_

Not only these two samples show very similar compositions (see chemical compositions in oxide wt%, Supplementary Table 1), but they also have compositions typical for mchr in diamonds^1^.

The results from the elastic geobarometry applied to diamond-mchr host-inclusion system in this work provide the first direct determination of provenance depth for a mchr-bearing diamond and new constraints on the thermal state and composition of the deep Siberian cratonic lithosphere at the time of diamond formation.

### Pressure-Volume-Temperature Equation of State of Reference mchr

**Figure 2:**
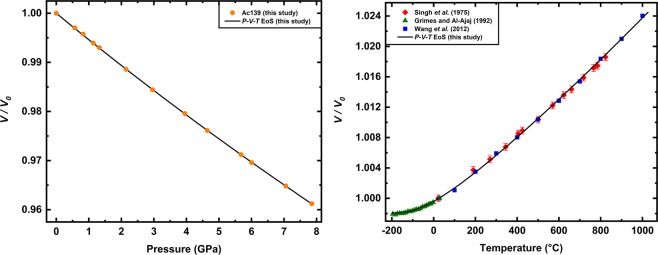
(**a**) Fractional unit-cell volume as a function of pressure measured in this work for Ac139 spinel. Uncertainties are smaller than symbols. (**b**) Fractional unit-cell volume as a function of temperature from literature^29–31^. The black line represents the fitting to the *P–V* and *T–V* data obtained from a BM2 EoS combined with the thermal-*P* model^28^. See text and Supplementary Table 3 for further details.

The best fit to the *P–V* data (Fig. 2a and Supplementary Table 2) for Ac139 is obtained for a second-order Birch-Murnaghan (BM2) EoS^27^, which gives the following parameters at room conditions: unit-cell volume *V*_0_ = 576.19(3) Å^3^, Reuss isothermal bulk modulus *K*_RT0_ = 182.8(6) GPa (first pressure derivative *K*_RT0_′ fixed to 4).

The full thermoelastic behavior of a mineral can be expressed through a *P–V–T* EoS that incorporates the concept of thermal pressure^28^. We therefore estimated the *P*–*V*–*T* EoS of Ac139 by combining the BM2 EoS at room conditions with the *T*–*V* data available in the literature for similar compositions. To constrain the high-*T* thermal behavior of our Ac139, we selected data^29^ obtained on a natural mchr spinel with composition Mg_0.58_Fe_0.42_Cr_1.52_Al_0.48_O_4_ to 823 °C, and those obtained^30^ on a synthetic magnesiochromite crystal (MgCr_2_O_4_) to 1000 °C. Figure 2b shows that the normalized unit-cell volumes as a function of *T* reported in these two studies are in very good agreement, suggesting that relatively large substitutions of Mg for Fe and of Cr for Al have negligible effects on the thermal behavior. Moreover, the literature data^30^ clearly show no cation order-disorder process, implying no order-disorder also for the mixed composition^29^. Since the chemical composition of sample Ac139 lies between the two chemical compositions investigated in the literature^29,30^, these two datasets can be considered representative also of its high-*T* behavior. To further constrain the thermal behavior, we also used the low-*T* data^31^ obtained on a synthetic polycrystalline MgAl_2_O_4_ spinel from −196 °C to room-*T*. The incorporation of the low-*T* data^31^, even referring to a chemical composition that is different from Ac139, does not affect the final fitting, but provides a further constraint (*i.e*. lower uncertainties) for the thermal EoS parameters. On a cubic mineral, like mchr, in which the thermal expansion and the axial moduli are equal in all directions, a thermal pressure model based on the quasi-harmonic approximation^28^ can be applied. Therefore, in order to determine the *P–V–T* EoS for mchr, we fitted *P–V* data for Ac139 and the selected *T–V* data from the literature by combining our BM2 EoS with the thermal pressure model^28^. The final *P–V–T* EoS parameters are reported in Supplementary Table 3. Considering that the bulk modulus is practically identical for both MgCr_2_O_4_ and FeCr_2_O_4_^25^ and that thermal expansion does not show significant variations for mixed compositions between magnesiochromite and chromite^29,30^, we are confident that our *P-V-T* EoS parameters can be applied to a wide range of mchr compositions, including the sample here investigated MgCr2.

Relatively to the effect of inversion of the spinel component on the inclusion volume at elevated temperature, it is negligible. In fact, for MgAl_2_O_4_ spinel from inversion (x) = 0, corresponding to an ideally ordered phase, to x = 0.29, corresponding to disorder at 1100 °C, volume change is only 0.3%, that is from 529.475 to 528.082 A^3^ (ref.^32^).

The effect of pressure is also negligible, as up to 7.5 GPa pressure has little or no influence on the degree of order in the MgAl_2_O_4_ spinel^33^.

Moreover, the influence of inversion on the elastic properties of MgAl_2_O_4_ spinel is also very limited. In fact, from x = 0 to x = 0.30, the decrease of bulk modulus *K*_RT0_ is about 1%, as shown by comparing results of density functional theory and Brillouin scattering technique^34^. Please note that all the considerations above refer to a pure MgAl_2_O_4_ spinel, whereas the MgAl_2_O_4_ component of our magnesiochromite inclusions is at maximum 13%.

### Diamond Residence Temperature and Depth of Formation of the Diamond-mchr pair

**Figure 3:**
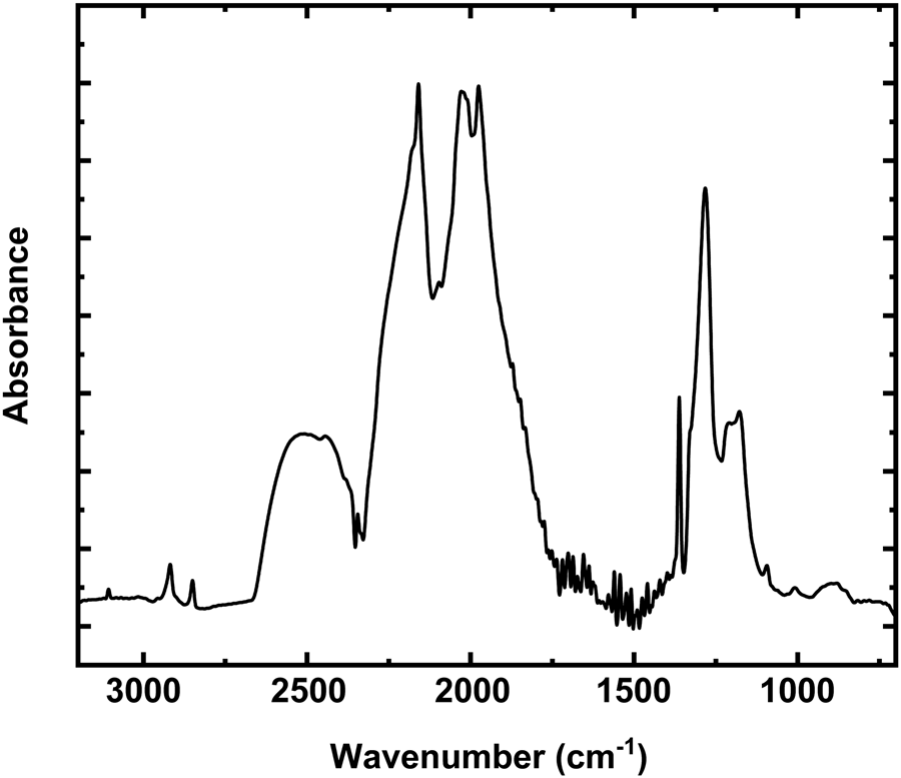
Representative FTIR spectrum of the diamond host (referred to Ud_4 measurement in Supplementary Table 4 and Supplementary Data 4).

The Fourier transform infrared (FTIR) measurements on the MgCr2 diamond host indicate an average N content (N_tot_) of 267 ppm and an aggregation of B defects percentage (%IaB) of 32% (Supplementary Table 4). In Fig. 3 we show one of the FTIR spectra we have collected on MgCr2 diamond host (in Supplementary Data 1 to 6 all FTIR spectra were available). These values are typical for Udachnaya diamonds^35^ and allow to classify the diamond as Type-IaAB. Based on the procedure reported in the Methods and taking into account the age of the host kimberlite (ca. 360 Ma^36^), our calculations indicate a mantle residence temperature, *T*_*res*_, of 1125(32) °C for a diamond age of 3.5 Ga and of 1140(33) °C for a diamond age of 2 Ga (Supplementary Table 4). These two ages cover the range of reported ages for Udachnaya diamonds^37–39^. The retrieved *T*_*res*_ values differ by less than their respective uncertainties and lie within the typical range of *T*_*res*_ for peridotitic diamonds from Udachnaya (ca. 1125–1175 °C, ref.^37^). Since a short residence at higher *T* during the early history of the diamond would have not produced significant effects on the final N-aggregation state, the estimated *T*_*res*_ of 1125–1140 °C can be considered as a minimum *T* estimate for diamond formation.

**Figure 4:**
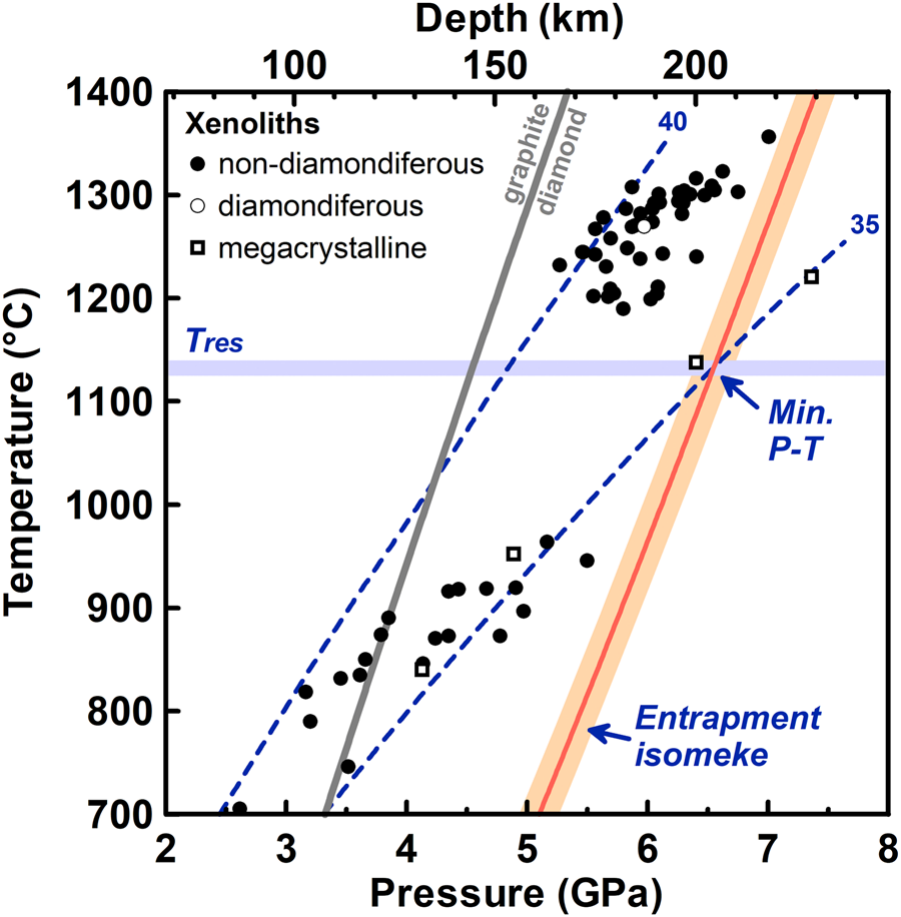
Pressure-temperature diagram showing the calculated entrapment isomeke for MgCr2 diamond-inclusion pair. *T*_*res*_ is the residence *T* based on diamond N-aggregation state. *P–T* estimates for Udachnaya peridotitic xenoliths (lherzolites) are mostly based on the combination of the orthopyroxene–garnet barometer^61^ with the two-pyroxene thermometer^62^. For the three highest-*P* megacrystalline peridotites (clinopyroxene-free) the orthopyroxene–garnet thermometer^63^ was used. Note that this thermometer is less reliable as it is sensitive to redox conditions^64^. Xenolith data are from the compilation of well-equilibrated lherzolites^63^, with additional data on megacrystalline peridotites^40,65^. Conductive geotherms for 35 and 40 mW/m^2^ are after ref.^66^; the diamond-graphite boundary is after ref.^67^.

The SC-XRD measurements performed on inclusion MgCr2 before and after its release from the diamond provided unit-cell volumes (*V*_0_) of 573.3(2) and 576.63(2) Å^3^, respectively. We used our *P–V* EoS (Supplementary Table 2) to convert the residual volume strain recorded by the inclusion still trapped in the host diamond into the hydrostatic component of the residual stress, *i.e*. the residual pressure (*P*_*inc*_), and obtained a value of 1.073(72) GPa at room conditions. The esd on *P*_*inc*_ was calculated by propagation of uncertainties on the EoS parameters (see Supplementary Table 3) and on *V*_0_ measurements. Using this *P*_*inc*_, the *P–V–T* EoS determined for the compositionally similar mchr Ac139, and the *P–V–T* EoS for diamond^7^, we can now calculate the entrapment conditions for the MgCr2 inclusion (shape of the inclusion and its distance from the diamond surface did not require further corrections^16^). Since both the host and the inclusion have cubic symmetry, we can apply the geobarometric model developed for isotropic systems and calculate the entrapment isomeke for the specific *P*_*inc*_ using the EosFit-Pinc software^10^. The entrapment isomeke is the line that defines all the possible entrapment conditions for a given *P*_*inc*_ (refs^6,8^). A specific entrapment pressure (*P*_*trap*_) can be obtained if the *T*_*trap*_ is independently constrained. Since the isomeke has a small ∂*P/*∂*T*, the *T*-dependency of the elastic geobarometer is low. Assuming for the MgCr2 inclusion a minimum *T*_*trap*_ equal to the estimated *T*_*res*_ of 1125–1140 °C, we obtain a minimum *P*_*trap*_ of 6.5(2) GPa (the uncertainty derives from the error propagation of all parameters used to calculate it), corresponding to a minimum depth of about 205(6) km (Fig. 4; the data used to plot the entrapment isomeke in figure are reported in Supplementary Table 5; the input files to run EosFit-Pinc software for our diamond - MgCr2 pair are available in Supplementary Data 7 and 8).

### The P-T Conditions of the Diamond-mchr System: Implications for the Mantle Under Udachnaya

Thermobarometric data obtained for mantle xenoliths from the Udachnaya kimberlite show relatively large scatter compared to many other cratonic mantle sections, making it difficult to outline a unique geotherm for this locality^40^ (Fig. 4). Existing data suggest that the mantle under Udachnaya experienced very cold conditions during at least part of its history, as testified by several peridotites and peridotitic garnets falling close to a ca. 35 mW/m^2^ conductive geotherm^40,41^. The lithosphere under Udachnaya extended to ca. 230 km depth at the time of kimberlite eruption, but the depth extension of the cold geotherm is poorly constrained due to scatter of *P–T* data. In fact, the deepest portion of the lithosphere records significant thermal perturbation, with the majority of high-*P* xenoliths (>5 GPa) being shifted to ca. 200–300 °C higher *T* (ref.^40^) (Fig. 4).

The estimated *P*_*trap*_ − *T*_*res*_ conditions for diamond - MgCr2 pair (6.5 GPa at ca. 1130 °C; see the *P* - *T* data relative to the isomeke in Fig. 4 provided between 700 and 1400 °C in Supplementary Data 9) fall right on the 35 mW/m^2^ geotherm (Fig. 4). This indicates that cold conditions have persisted for a long geological time in the Udachnaya lithosphere to at least 200 km depth. Heating processes shortly before or during kimberlite eruption might lead to overestimation of the real *T*_*res*_ in the mantle and, consequently, of *P*_*trap*_. However, this effect cannot be significant, otherwise *P–T* estimates along the entrapment isomeke would become incompatible with even the coldest cratonic geotherm (Fig. 4). We cannot exclude that our diamond has formed during a *short* thermal pulse at conditions somewhat hotter than the 35 mW/m^2^ geotherm, as this would not be recorded in its N-aggregation state^1^. In this case, however, the real *P*_*trap*_ would be slightly higher (*e.g*. ca. 7 GPa for a *T*_*trap*_ of 1300 °C) and the depth extension of the long-lived cold geotherm would only be greater (i.e., to ca. 1130 °C and 7 GPa, or 220 km). Much higher *P*_*trap*_, however, would imply long-term residence at conditions, again, too cold for a cratonic lithosphere (Fig. 4). For the same reasons, significant *P*_*trap*_ underestimation due to plastic deformation and *P*_*inc*_ relaxation can be excluded. Disregarding the inevitable small discrepancies between *P–T* estimates obtained with different thermobarometric methods, our results provide independent confirmation of literature hypothesis^41^, based on scanty xenolith (megacrystalline peridotites) and garnet xenocryst data, that a cold 35 mW/m^2^ geotherm originally extended to ca. 1200 °C.

Abundant experimental data and thermodynamic modeling^2,42–46^ showed that the stability of mchr in peridotite is strongly dependent on the peridotite ‘fertility’ and that in the garnet–spinel stability field Cr#_spinel_ increases rapidly with increasing *P* and slightly with decreasing *T*. At given *P–T*, Cr#_spinel_ depends on the spinel modal abundance and is thus lower in more spinel-rich, more depleted peridotites^2^. Thermodynamic modeling in natural peridotitic systems^2^ predicts that, along a 35 mW/m^2^ geotherm in very depleted harzburgite with bulk Cr# = 0.32, spinel is only stable to 5.5 GPa and 1000 °C, where it reaches a Cr#_spinel_ of ca. 0.9. These conditions are not compatible with our *P–T* estimates (Fig. 4). Along hotter geotherms or in less depleted peridotites, the stability of spinel would be restricted to even lower *P*. This suggests that our mchr belonged to a more depleted, possibly dunitic and spinel-rich assemblage. The persistence of a Cr# ∼ 0.9 (namely, 0.86) in our mchr at 6.5 GPa further supports this hypothesis.

Compared with classical xenolith geothermobarometry, diamond elastic geothermobarometry has a considerable potential to yield ancient lithospheric thermal states, because diamonds can be very old, and their inclusions remain exceptionally preserved from chemical exchanges with surrounding minerals, fluids and melts even under dramatically changing physico-chemical conditions. With our unprecedented application of elastic geothermobarometry to a mchr inclusion in a gem-quality octahedral diamond from the Udachnaya kimberlite, we provided evidence that cold conditions corresponding to a ca. 35 mW/m^2^ geotherm persisted for billions of years to a depth of at least 200 km in the Siberian craton lithosphere, and that at this depth the lithosphere was, at least locally, ultra-depleted.

## Methods

### Samples

We have studied two mchr samples: the first sample was a released crystal used to determine the EoS of mchr, while the second sample was still included within a diamond and was used to determine the depth of formation of the diamond–mchr host–inclusion pair using the thermoelastic properties determined on the first one. The first mchr, labeled Ac139, is a mchr single crystal with a size of 42 × 25 × 15 μm^3^ and high crystal quality, *i.e*. sharp diffraction profiles and absence of twinning and inclusions. It was extracted from a lithospheric diamond from the Aikhal kimberlite (Siberia, Russia) by crushing the diamond and then studied by *in-situ* high-pressure single-crystal X-ray diffraction. The second mchr, labeled MgCr2, was included in a gemstone octahedral diamond (longest dimension = 2.15 mm) from the Udachnaya kimberlite, Siberia, Russia (Fig. 1), and belongs to the same set of diamonds previously studied^22,47^. The MgCr2 inclusion was alone within its host and located in intermediate position between the gravimeter center and external surface of the diamond. Its morphology is nearly equant cube-octahedral, with an average crystal radius of 0.34 mm, and no optically visible fractures were observed. The mchr inclusion was extracted from its host by crushing the diamond only after a first set of *in-situ* X-ray analyses.

### Electron microprobe analyses

Chemical analyses were carried out on sample Ac139 using a CAMECA SX50 electron microprobe (WDS mode, 20 kV, 20 nA, 2 μm beam diameter) at CNR-IGG of Padova. Standards used were Kakanui pyrope (New Zealand) from the Smithsonian Museum (TAP analyser crystal, Mg*K*α element emission line); Amelia albite (Virginia) (TAP, Na*K*α); diopside (TAP, Si*K*α); Al_2_O_3_ (TAP, Al*K*α); MnTiO_3_ (PET, Ti*K*α; LIF, Mn*K*α); Cr_2_O_3_ (LIF, Cr*K*α); Fe_2_O_3_ (LIF, Fe*K*α); sphalerite (LIF, Zn*K*α); NiO (LIF, Ni*K*α). Raw data were reduced with the PAP-type correction software provided by CAMECA. The resulting oxides wt% are reported in Supplementary Table 1. Chemical analyses on sample MgCr2 were carried out by standardless EDS analysis on a FEG scanning-electron microscope, using an Oxford Instruments SDD detector and the AZtec TruQ software installed at the Oxford Instruments in Wiessbaden (Germany).

### Fourier transform Infrared spectroscopy

Fourier Transform Infrared (FTIR) were acquired using a Thermo Scientific Nicolet Centaurμs FT-IR Microscope (located at the Department of Industrial Engineering, University of Padova) operated via OMNIC software. The spectra were collected in the 4000–650 cm^−1^ wavelength range, with 64 scans of sample exposure and 0.5 cm^−1^ of spectral resolution. The background spectra were run for 120 s before the analyses and the sample spectra were subtracted by them. The measurements were performed on a diamond plate collected from the diamond crushed material after the MgCr2 crystal release. The plate was mounted in wax to allow the analyses in transmission mode. We acquired different FTIR spectra over six different locations on the plate with an FTIR spot of about 100 μm, covering most of its area. The absorbance spectra were processed using DiaMap^48,49^ to obtain the N content and aggregation state^50^. This program fits the individual N absorption band and deconvolutes the spectrum in the 1001 to 1350 cm^−1^ range. Then, the concentrations of N impurities aggregated in pairs (A defects) and of N impurities aggregated in clusters plus vacancy (B defects) were calculated using literature absorption coefficients^51,52^. Errors in calculation of N content and aggregation state are considered of ±10%^48^ and depend on the quality of the FTIR spectra. The original FTIR spectra collected in these analyses are reported in the supplementary data (1 to 6), while the data on N content and aggregation state are reported in Supplementary Table 4. A representative FTIR spectrum for the diamond host of the sample MgCr2 (data from such spectrum are relative to Ud_4 in Supplementary Table 4) is shown in Fig. 2.

We used the constants from the literature^53,54^ (*i.e. E*/*R* = 81160 K; ln(*A*) = 12.59; where: *E* = activation energy; *R* = gas constant; *A* = Arrhenius constant) to calculate the residence temperature, *T*_*res*_, as a function of time for the MgCr2 diamond. The age of our diamond is unknown, but previous geochronological work on Udachnaya diamonds suggested two major peaks of diamond formation, one at 3.1–3.5 Ga (Re-Os dating on sulphide inclusions^35,36^) and one at ca. 2 Ga (Sm-Nd dating on garnet inclusions^37^).

The calculations indicate a mantle residence temperature, *T*_*res*_, of 1125(32) °C for the oldest age (3.5 Ga) and of 1140(33) °C for the youngest age (2 Ga). Uncertainties reported in parentheses (estimated standard deviations, esd) were calculated from the propagation of the uncertainties on the FTIR measurement and on constants taken from literature^53,54^.

### X-ray diffraction measurements

*In-situ* high-pressure single-crystal X-ray diffraction (SC-XRD) experiments were performed on mchr Ac139, in order to determine its thermoelastic properties and *Pressure* − *Volume* Equation of State. The crystal was loaded in an ETH-type diamond-anvil cell (DAC) equipped with Be-backing plates^55^. We used a steel gasket pre-indented to a thickness of about 90 μm with a spark-eroded hole of 250 μm in diameter and a mixture of methanol:ethanol with ratio 4:1 to ensure hydrostatic pressures up to the maximum *P* reached in this work (7.852 GPa) (it is well established that for such pressure values the mixture used behaves hydrostatically^56^). The diameter of the diamond culet was 600 μm. The sample was loaded together with a single crystal of quartz used as an internal pressure standard^57^. The unit-cell edge was measured on a four-circle STOE STADI IV diffractometer equipped with a point detector and controlled by the SINGLE software^58^ and installed at the Department of Geosciences, University of Padova. The SINGLE software is based on the 8-position centering method^59^ and vector least-squares refinement of the unit-cell parameters^60^. The data were acquired using Mo*K*α radiation at 50 kV and 40 mA. Unit-cell parameters were determined from the centering of an average of 19 reflections for each high-pressure measurement, in the 2*θ* range between 8° and 34°. The unit-cell edges and unit-cell volumes at different pressures are reported in Supplementary Table 2.

The determination of the residual pressure (*P*_*inc*_) on inclusion MgCr2 was obtained by comparing the unit-cell parameters measured on the crystal when still trapped in its host and, then, in air after extraction from the diamond. The SC-XRD measurements were performed by using a Rigaku-Oxford Diffraction Supernova diffractometer equipped with a micro-source Mo*K*α radiation, working at 50 kV and 0.8 mA and with a sample-to-detector distance of 68 mm, and a Pilatus 200 K Dectris detector. The diffractometer was installed at the Department of Geosciences, University of Padova. The unit-cell edges before and after the release from the diamond host were measured by collecting data up to 2*θ* = 80°, using a thin slicing in *ω* of 0.1° (see values at the section Diamond residence temperature and depth of formation of the diamond-mchr pair).

## Supplementary information

Supplementary dataset n. 1

Supplementary dataset n. 2

Supplementary dataset n. 3

Supplementary dataset n. 4

Supplementary dataset n. 5

Supplementary dataset n. 6

Supplementary dataset n. 7

Supplementary dataset n. 8

Supplementary dataset n. 9

Supplementary materials

## References

[1] Stachel T, Harris JW (2008). The origin of cratonic diamonds - Constraints from mineral inclusions. Ore Geol. Rev..

[2] Ziberna L, Klemme S, Nimis P (2013). Garnet and spinel in fertile and depleted mantle: insights from thermodynamic modelling. Contrib. Mineral. Petr..

[3] Rosenfeld JL, Chase AB (1961). Pressure and temperature of crystallization from elastic effects around solid inclusions in minerals?. Am. J. Sci..

[4] Adams HG, Cohen LH, Rosenfeld JL (1975). Solid inclusion piezothermometry I: comparison dilatometry. Am. Mineral..

[5] Angel RJ, Gonzalez-Platas J, Alvaro M (2014). EosFit7c and a Fortran module (library) for equation of state calculations. Z. Krist..

[6] Angel RJ, Mazzucchelli ML, Alvaro M, Nimis P, Nestola F (2014). Geobarometry from host-inclusion systems: the role of elastic relaxation. Am. Mineral..

[7] Angel RJ, Alvaro M, Nestola F, Mazzucchelli ML (2015). Diamond thermoelastic properties and implications for determining the pressure of formation of diamond-inclusion systems. Russ. Geol. Geophys..

[8] Angel RJ, Nimis P, Mazzucchelli ML, Alvaro M, Nestola F (2015). How large are departures from lithostatic pressure? Constraints from host-inclusion elasticity. J. Metamorph. Geol..

[9] Angel RJ, Alvaro M, Miletich R, Nestola F (2017). A simple and generalised P–T–V EoS for continuous phase transitions, implemented in EosFit and applied to quartz. Contrib. Mineral. Petrol..

[10] Angel RJ, Mazzucchelli ML, Alvaro M, Nestola F (2017). EosFit-Pinc: A simple GUI for host-inclusion elastic thermobarometry. Am. Mineral..

[11] Angel RJ (2019). Stress, strain and Raman shifts. Z. Krist..

[12] Milani S (2015). Diamond–garnet geobarometry: The role of garnet compressibility and expansivity. Lithos.

[13] Milani S (2017). Thermoelastic behaviour of grossular garnets at high pressures and temperatures. Am. Mineral..

[14] Anzolini C (2016). Depth of formation of CaSiO_3_-walstromite included in super-deep diamonds. Lithos.

[15] Anzolini C (2018). Depth of formation of super-deep diamonds: Raman barometry of CaSiO_3_-walstromite inclusions. Am. Mineral..

[16] Mazzucchelli ML (2018). Elastic geothermobarometry: corrections for the geometry of the host-inclusion system. Geology.

[17] Nestola F (2018). Toward a robust elastic geobarometry of kyanite inclusions in eclogitic diamonds. J. Geophys. Res..

[18] Angel RJ, Alvaro M, Nestola F (2017). 40 years of mineral elasticity: a critical review and a new parameterisation of equations of state for mantle olivines and diamond inclusions. Phys. Chem. Miner..

[19] Campomenosi N (2018). How geometry and anisotropy affect residual strain in host-inclusion systems: Coupling experimental and numerical approaches. Am. Mineral..

[20] Murri M (2018). Raman elastic geobarometry for anisotropic mineral inclusions. Am. Mineral..

[21] Murri M, Alvaro M, Angel RJ, Prencipe M, Mihailova BD (2019). The effects of non-hydrostatic stress on the structure and properties of alpha-quartz. Phys. Chem. Miner..

[22] Nimis P (2016). First evidence of hydrous silicic fluid films around solid inclusions in gem-quality diamonds. Lithos.

[23] Smith EM (2016). Large gem diamonds from metallic liquid in Earth’s deep mantle. Science.

[24] Yong W, Botis S, Shieh SR, Shi W, Withers AC (2012). Pressure-induced phase transition study of magnesiochromite (MgCr_2_O_4_) by Raman spectroscopy and X-ray diffraction. Phys. Earth Planet. In..

[25] Nestola F (2014). Pressure-volume equation of state for chromite and magnesiochromite: A single-crystal X-ray diffraction investigation. Am. Mineral..

[26] Zhang YY, Liu X, Xiong Z, Zhang Z (2016). Compressional behavior of MgCr_2_O_4_ spinel from first-principles simulation. Sci. China Earth Sci..

[27] Birch F (1947). Finite elastic strain of cubic crystals. Phys. Rev..

[28] Holland TJB, Powell R (2011). An improved and extended internally consistent thermodynamic dataset for phases of petrological interest, involving a new equation of state for solids. J. Metamorph. Geol..

[29] Singh HP, Simmons G, McFarlin PF (1975). Thermal expansion of natural spinel, ferroan gahnite, magnesiochromite and synthetic spinel. Acta Crystall. A.

[30] Wang S, Liu X, Fei Y, He Q, Wang H (2012). *In situ* high-temperature powder X-ray diffraction study on the spinel solid solutions (Mg1− xMnx)Cr_2_O_4_. Phys. Chem. Miner..

[31] Grimes NW, Al-Ajaj EA (1992). Low-temperature thermal expansion of spinel. J. Phys..

[32] Andreozzi GB, Princivalle F, Skogby H, Della Giusta A (2000). Cation ordering and structural variations with temperature in MgAl_2_O_4_ spinel: An X-ray single-crystal study. Am. Mineral..

[33] Nestola F (2007). Comparative compressibility and structural behavior of spinel MgAl2O4 at high pressures: The independency on the degree of cation order. Am. Mineral..

[34] Nunez-Valdez M (2018). Reexploring the cation ordering and magnetic cation substitution effects on the elastic anisotropy of aluminum spinels. J. Appl. Phys..

[35] Palot M (2013). Multiple Growth Episodes or Prolonged Formation of Diamonds? Inferences from Infrared Absorption Data. J. Geol. Soc. India..

[36] Kinny PD, Griffin BJ, Heaman LM, Brakhfogel FF, Spetsius ZV (1997). SHRIMP U-Pb ages of perovskite from Yakutian kimberlites. Russian Geol. Geophys..

[37] Richardson SH, Harris JW (1997). Antiquity of peridotitic diamonds from the Siberian craton. Earth Planet. Sci. Lett..

[38] Pearson DG, Shirey SB, Bulanova GP, Carlson RW, Milledge HJ (1999). Re-Os isotope measurements of single sulfide inclusions in a Siberian diamond and its nitrogen aggregation systematics. Geochim. Cosmochim. Acta.

[39] Pearson, D. G. *et al*. (eds) The P. H. Nixon Volume—Proceedings of the Seventh International Kimberlite Conference, Cape Town, Red Roof Design, Cape Town, pp 637–643 (1999).

[40] Doucet LS, Ionov DA, Golovin AV (2013). The origin of coarse garnet peridotites in cratonic lithosphere: new data on xenoliths from the Udachnaya kimberlite, central Siberia. Contrib. Mineral. Petr..

[41] Griffin WL (1996). Thermal state and composition of the lithospheric mantle beneath the Daldyn kimberlite field, Yakutia. Tectonophysics.

[42] O’Neill HSC (1981). The transition between spinel lherzolite and garnet lherzolite, and its use as a geobarometer. Contrib. Mineral. Petrol..

[43] Nickel, K. G. Garnet-pyroxene equilibria in the system SMACCR (SiO_2_–MgO–Al_2_O_3_–CaO–Cr_2_O_3_): the Cr-geobarometer. In Ross, J., Jaques, A. L., Ferguson, J., Green, D. H., O’Reilly, S. Y., Danchin, R. V., Janse, A. J. A. (eds) *Kimberlites and related rocks, vol. 2, their mantle/crust setting, diamonds and diamond exploration.* Geological Society of Australia Special Publication 14. Blackwell Scientific, Victoria, pp 901–912 (1989).

[44] Webb SAC, Wood BJ (1986). Spinel-pyroxene-garnet relationships and their dependence on Cr/Al ratio. Contrib. Mineral. Petrol..

[45] Doroshev AM, Brey GP, Girnis AV, Turkin AI, Kogarko LN (1997). Pyrope-knorringite garnets in the Earth’s mantle: Experiments in the MgO-Al_2_O_3_-SiO_2_-Cr_2_O_3_ system. Russ. Geol. Geophys..

[46] Klemme S, Ivanic TJ, Connolly JAD, Harte B (2009). Thermodynamic modelling of Cr-bearing garnets with implications for diamond inclusions and peridotite xenoliths. Lithos.

[47] Nimis P (2019). Crystallographic orientations of magnesiochromite inclusions in diamonds: what do they tell us?. Contrib. Mineral. Petr..

[48] Howell D (2012). μ-FTIR mapping: distribution of impurities in different types of diamond growth. Diam. Relat. Mater..

[49] Howell D (2012). Platelet development in cuboid diamonds: insights from micro-FTIR mapping. Contrib. Mineral. Petr..

[50] Breeding CM, Shigley JE (2009). The “Type” Classification system of diamonds and its importance in gemology. Gems Gemol..

[51] Boyd SR, Kiflawi I, Woods GS (1994). The relationship between infrared absorption and the A defect concentration in diamond. Philos. Mag..

[52] Boyd SR, Kiflawi I, Woods GS (1995). Infrared absorption by the B nitrogen aggregate in diamond. Philos. Mag..

[53] Leahy K, Taylor WR (1997). The influence of the Glennie domain deep structure on the diamonds in Saskatchewan kimberlites. Geol. Geofiz..

[54] Taylor WR, Jaques AL, Ridd M (1990). Nitrogen-defect aggregation characteristics of some Australasian diamonds: Time-temperature constraints on the source regions of pipe and alluvial diamonds. Am. Mineral..

[55] Miletich R, Allan DR, Kuhs WF (2000). High-pressure single-crystal techniques. Rev. Mineral. Geochem..

[56] Angel RJ, Bujak M, Zhao J, Gatta GD, Jacobsen SD (2007). Effective hydrostatic limits of pressure media for high-pressure crystallographic studies. J. Appl. Crystallogr..

[57] Scheidl KS (2016). Extending the single-crystal quartz pressure gauge up to hydrostatic pressure of 19 GPa. J. Appl. Crystallogr..

[58] Angel RJ, Finger L (2011). SINGLE: a program to control single-crystal diffractometers. J. Appl. Crystallogr..

[59] King HE, Finger LW (1979). Diffracted beam crystal centering and its application to high-pressure crystallography. J. Appl. Crystallogr..

[60] Angel RJ, Downs RT, Finger LW (2000). High-temperature–high-pressure diffractometry. Rev. Mineral. Geochem..

[61] Nickel KG, Green DH (1984). Empirical geothermobarometry for garnet peridotites and implications for the nature of the lithosphere, kimberlites and diamonds. Earth Planet. Sci. Lett..

[62] Taylor WR (1998). An experimental test of some geothermometer and geobarometer formulations for upper mantle peridotites with application to the thermobarometry of fertile lherzolite and garnet websterite. Neues J. Mineral. Abh..

[63] Nimis P, Grütter H (2010). Internally consistent geothermometers for garnet peridotites and pyroxenites. Contrib. Mineral. Petrol..

[64] Nimis P, Goncharov A, Ionov DA, McCammon C (2015). Fe^3+^ partitioning systematics between orthopyroxene and garnet in mantle peridotite xenoliths and implications for thermobarometry of oxidized and reduced mantle rocks. Contrib. Mineral. Petrol..

[65] Pokhilenko, N. P., Pearson, D. G., Boyd, F. R. & Sobolev, N. V. Megacrystalline dunites and peridotites: hosts for Siberian diamonds. *Ann. Rep. Dir. Geophys. Lab. Carn. Inst. Wash*., 11–18 (1991).

[66] Hasterok D, Chapman DS (2011). Heat production and geotherms for the continental lithosphere. Earth Planet. Sc. Lett..

[67] Day HW (2012). A revised diamond-graphite transition curve. Am. Mineral..

