# Supplementary material for: Diamond-inclusion system recording old deep lithosphere conditions at Udachnaya (Siberia)
Source: Sci Rep. 2019 Aug 29;9:12586. doi: 10.1038/s41598-019-48778-x (PMC6715805; doi:10.1038/s41598-019-48778-x)
Supplement: Supplementary file 7 — Supplementary dataset n. 7 [file 41598_2019_48778_MOESM7_ESM.docx]

**Diamond-inclusion system recording old deep lithosphere conditions at Udachnaya (Siberia)**

**Nestola Fabrizio^1^, Zaffiro Gabriele^2^, Mazzucchelli Mattia L.^2^, Nimis Paolo^1^, Andreozzi Giovanni B.^3^, Periotto Benedetta^1^, Princivalle Francesco^4^, Lenaz Davide^4^, Secco Luciano^1^, Pasqualetto Leonardo^1^, Logvinova Alla M.^5,6^, Sobolev Nikolay V.^5,6^, Lorenzetti Alessandra^7^, Harris Jeffrey W.^8^**

***********************************************************************************************************************

* *

* EOSFIT7c *

* *

* Authors: RJ ANGEL, J GONZALEZ-PLATAS, M ALVARO *

* Version date: 20-JULY-2016 *

* Version: 7.34 *

* *

* *

* INCLUDING LEAST SQUARES BY I.G. WOOD & J. PIPER AND USING THE CrysFML EOS MODULE. *

* *

* EosFit7c: Angel, Gonzalez-Platas, Alvaro (2014) Zeitschrift fuer Kristallographie, 229:405-419. *

* Phase Transitions: Angel et al (2016) Contribs Min Pet, submitted. *

* Fitting Moduli: Milani et al. (2016) Amer Min, submitted. *

* *

* Development supported by ERC grant 307322, INDIMEDEA to F. Nestola, *

* SIR-MIUR grant RBSI140351, MILE DEEp, to M. Alvaro, and *

* Programa de Redes de Excelencia MAT2015-71070-REDC to J. Gonzalez-Platas *

* TEST VERSION NOT FOR GENERAL RELEASE *

* *

* *

* Current date and time: 25-JUL-2016 17:51 *

* *

***********************************************************************************************************************

EosFit parameter file

This is a fixed-format file. If you edit this file, make sure you do not move anything!

It is safer to change the parameters by loading the file to EosFit, and saving the file after making changes

____________________________________________________________________________________________________________

Title =PVT-EOS for diamond

Comment =Data obtained as described in Angel et al. (2014), Russian geology Geophysics, submitted

Comment =Eos saved on 6-JUN-2014 15:10

Model = 5, (Tait)

Order = 2

Thermal = 6, (HP Thermal Pres)

Trans = 0, (none)

Shear = 1, (Polynomial)

Type = Volume

Pref = 0.00000

Tref = 300.00000

Pscale =GPa

Vscale =

Stoich = 1.00000

Param = 1 1.000000 (V0 , Reference pressure volume: units as volume data)

Param = 2 444.000000 (K0 , Bulk modulus: units are GPa)

Param = 3 4.000000 (Kp , dK/dP: dimensionless)

Param = 4 -0.009009 (Kpp , d2K/dP2: units are inverse GPa)

Param = 5 0.000000

Param = 6 0.000000

Param = 7 0.000000

Param = 8 0.000000

Param = 9 0.000000

Param =10 0.267194 (alph0, Constant of thermal expansion at Tref x10^5 K^-1)

Param =11 1500.000000 (Th_E , Einstein temperature in K)

Param =12 0.000000

Param =13 0.000000 (Gamm0, Gruneisen parameter at Tref,Pref)

Param =14 0.000000 (q , Gruneisen power law in V/V0)

Param =15 0.000000

Param =16 0.000000

Param =17 0.000000

Param =18 0.000000

Param =19 0.000000

Param =20 0.000000

Param =21 0.000000

Param =22 0.000000

Param =23 0.000000

Param =24 0.000000

Param =25 0.000000

Param =26 0.000000

Param =27 0.000000

Param =28 0.000000

Param =29 0.000000

Param =30 535.000000 (G0 , Shear modulus at Pref, Tref, in pressure units)

Param =31 0.000000 (dG/dP, Pressure derivative of shear modulus: no units)

Param =32 0.000000 (d2G/ , 2nd Pressure derivative of shear modulus: P^-1)

Param =33 0.000000 (d3G/ , 3rd Pressure derivative of shear modulus: P^-2)

Param =34 0.000000 (dG/dT, Temperature derivative of shear modulus)

Param =35 0.000000

Param =36 0.000000

Param =37 0.000000

Param =38 0.000000

Param =39 0.000000

Param =40 0.000000

Variance-Covariance matrix=

0.73367E-06 0.00000E+00 0.00000E+00 0.00000E+00 0.00000E+00 0.00000E+00 0.00000E+00 0.00000E+00 0.00000E+00-0.18561E-06 0.00000E+00 0.00000E+00 0.00000E+00 0.00000E+00 0.00000E+00 0.00000E+00 0.00000E+00 0.00000E+00 0.00000E+00 0.00000E+00 0.00000E+00 0.00000E+00 0.00000E+00 0.00000E+00 0.00000E+00 0.00000E+00 0.00000E+00 0.00000E+00 0.00000E+00 0.00000E+00 0.00000E+00 0.00000E+00 0.00000E+00 0.00000E+00 0.00000E+00 0.00000E+00 0.00000E+00 0.00000E+00 0.00000E+00 0.00000E+00

0.00000E+00 0.40000E+01 0.00000E+00 0.00000E+00 0.00000E+00 0.00000E+00 0.00000E+00 0.00000E+00 0.00000E+00 0.00000E+00 0.00000E+00 0.00000E+00 0.00000E+00 0.00000E+00 0.00000E+00 0.00000E+00 0.00000E+00 0.00000E+00 0.00000E+00 0.00000E+00 0.00000E+00 0.00000E+00 0.00000E+00 0.00000E+00 0.00000E+00 0.00000E+00 0.00000E+00 0.00000E+00 0.00000E+00 0.00000E+00 0.00000E+00 0.00000E+00 0.00000E+00 0.00000E+00 0.00000E+00 0.00000E+00 0.00000E+00 0.00000E+00 0.00000E+00 0.00000E+00

0.00000E+00 0.00000E+00 0.00000E+00 0.00000E+00 0.00000E+00 0.00000E+00 0.00000E+00 0.00000E+00 0.00000E+00 0.00000E+00 0.00000E+00 0.00000E+00 0.00000E+00 0.00000E+00 0.00000E+00 0.00000E+00 0.00000E+00 0.00000E+00 0.00000E+00 0.00000E+00 0.00000E+00 0.00000E+00 0.00000E+00 0.00000E+00 0.00000E+00 0.00000E+00 0.00000E+00 0.00000E+00 0.00000E+00 0.00000E+00 0.00000E+00 0.00000E+00 0.00000E+00 0.00000E+00 0.00000E+00 0.00000E+00 0.00000E+00 0.00000E+00 0.00000E+00 0.00000E+00

0.00000E+00 0.00000E+00 0.00000E+00 0.00000E+00 0.00000E+00 0.00000E+00 0.00000E+00 0.00000E+00 0.00000E+00 0.00000E+00 0.00000E+00 0.00000E+00 0.00000E+00 0.00000E+00 0.00000E+00 0.00000E+00 0.00000E+00 0.00000E+00 0.00000E+00 0.00000E+00 0.00000E+00 0.00000E+00 0.00000E+00 0.00000E+00 0.00000E+00 0.00000E+00 0.00000E+00 0.00000E+00 0.00000E+00 0.00000E+00 0.00000E+00 0.00000E+00 0.00000E+00 0.00000E+00 0.00000E+00 0.00000E+00 0.00000E+00 0.00000E+00 0.00000E+00 0.00000E+00

0.00000E+00 0.00000E+00 0.00000E+00 0.00000E+00 0.00000E+00 0.00000E+00 0.00000E+00 0.00000E+00 0.00000E+00 0.00000E+00 0.00000E+00 0.00000E+00 0.00000E+00 0.00000E+00 0.00000E+00 0.00000E+00 0.00000E+00 0.00000E+00 0.00000E+00 0.00000E+00 0.00000E+00 0.00000E+00 0.00000E+00 0.00000E+00 0.00000E+00 0.00000E+00 0.00000E+00 0.00000E+00 0.00000E+00 0.00000E+00 0.00000E+00 0.00000E+00 0.00000E+00 0.00000E+00 0.00000E+00 0.00000E+00 0.00000E+00 0.00000E+00 0.00000E+00 0.00000E+00

0.00000E+00 0.00000E+00 0.00000E+00 0.00000E+00 0.00000E+00 0.00000E+00 0.00000E+00 0.00000E+00 0.00000E+00 0.00000E+00 0.00000E+00 0.00000E+00 0.00000E+00 0.00000E+00 0.00000E+00 0.00000E+00 0.00000E+00 0.00000E+00 0.00000E+00 0.00000E+00 0.00000E+00 0.00000E+00 0.00000E+00 0.00000E+00 0.00000E+00 0.00000E+00 0.00000E+00 0.00000E+00 0.00000E+00 0.00000E+00 0.00000E+00 0.00000E+00 0.00000E+00 0.00000E+00 0.00000E+00 0.00000E+00 0.00000E+00 0.00000E+00 0.00000E+00 0.00000E+00

0.00000E+00 0.00000E+00 0.00000E+00 0.00000E+00 0.00000E+00 0.00000E+00 0.00000E+00 0.00000E+00 0.00000E+00 0.00000E+00 0.00000E+00 0.00000E+00 0.00000E+00 0.00000E+00 0.00000E+00 0.00000E+00 0.00000E+00 0.00000E+00 0.00000E+00 0.00000E+00 0.00000E+00 0.00000E+00 0.00000E+00 0.00000E+00 0.00000E+00 0.00000E+00 0.00000E+00 0.00000E+00 0.00000E+00 0.00000E+00 0.00000E+00 0.00000E+00 0.00000E+00 0.00000E+00 0.00000E+00 0.00000E+00 0.00000E+00 0.00000E+00 0.00000E+00 0.00000E+00

0.00000E+00 0.00000E+00 0.00000E+00 0.00000E+00 0.00000E+00 0.00000E+00 0.00000E+00 0.00000E+00 0.00000E+00 0.00000E+00 0.00000E+00 0.00000E+00 0.00000E+00 0.00000E+00 0.00000E+00 0.00000E+00 0.00000E+00 0.00000E+00 0.00000E+00 0.00000E+00 0.00000E+00 0.00000E+00 0.00000E+00 0.00000E+00 0.00000E+00 0.00000E+00 0.00000E+00 0.00000E+00 0.00000E+00 0.00000E+00 0.00000E+00 0.00000E+00 0.00000E+00 0.00000E+00 0.00000E+00 0.00000E+00 0.00000E+00 0.00000E+00 0.00000E+00 0.00000E+00

0.00000E+00 0.00000E+00 0.00000E+00 0.00000E+00 0.00000E+00 0.00000E+00 0.00000E+00 0.00000E+00 0.00000E+00 0.00000E+00 0.00000E+00 0.00000E+00 0.00000E+00 0.00000E+00 0.00000E+00 0.00000E+00 0.00000E+00 0.00000E+00 0.00000E+00 0.00000E+00 0.00000E+00 0.00000E+00 0.00000E+00 0.00000E+00 0.00000E+00 0.00000E+00 0.00000E+00 0.00000E+00 0.00000E+00 0.00000E+00 0.00000E+00 0.00000E+00 0.00000E+00 0.00000E+00 0.00000E+00 0.00000E+00 0.00000E+00 0.00000E+00 0.00000E+00 0.00000E+00

-0.18561E-06 0.00000E+00 0.00000E+00 0.00000E+00 0.00000E+00 0.00000E+00 0.00000E+00 0.00000E+00 0.00000E+00 0.12745E-06 0.00000E+00 0.00000E+00 0.00000E+00 0.00000E+00 0.00000E+00 0.00000E+00 0.00000E+00 0.00000E+00 0.00000E+00 0.00000E+00 0.00000E+00 0.00000E+00 0.00000E+00 0.00000E+00 0.00000E+00 0.00000E+00 0.00000E+00 0.00000E+00 0.00000E+00 0.00000E+00 0.00000E+00 0.00000E+00 0.00000E+00 0.00000E+00 0.00000E+00 0.00000E+00 0.00000E+00 0.00000E+00 0.00000E+00 0.00000E+00

0.00000E+00 0.00000E+00 0.00000E+00 0.00000E+00 0.00000E+00 0.00000E+00 0.00000E+00 0.00000E+00 0.00000E+00 0.00000E+00 0.00000E+00 0.00000E+00 0.00000E+00 0.00000E+00 0.00000E+00 0.00000E+00 0.00000E+00 0.00000E+00 0.00000E+00 0.00000E+00 0.00000E+00 0.00000E+00 0.00000E+00 0.00000E+00 0.00000E+00 0.00000E+00 0.00000E+00 0.00000E+00 0.00000E+00 0.00000E+00 0.00000E+00 0.00000E+00 0.00000E+00 0.00000E+00 0.00000E+00 0.00000E+00 0.00000E+00 0.00000E+00 0.00000E+00 0.00000E+00

0.00000E+00 0.00000E+00 0.00000E+00 0.00000E+00 0.00000E+00 0.00000E+00 0.00000E+00 0.00000E+00 0.00000E+00 0.00000E+00 0.00000E+00 0.00000E+00 0.00000E+00 0.00000E+00 0.00000E+00 0.00000E+00 0.00000E+00 0.00000E+00 0.00000E+00 0.00000E+00 0.00000E+00 0.00000E+00 0.00000E+00 0.00000E+00 0.00000E+00 0.00000E+00 0.00000E+00 0.00000E+00 0.00000E+00 0.00000E+00 0.00000E+00 0.00000E+00 0.00000E+00 0.00000E+00 0.00000E+00 0.00000E+00 0.00000E+00 0.00000E+00 0.00000E+00 0.00000E+00

0.00000E+00 0.00000E+00 0.00000E+00 0.00000E+00 0.00000E+00 0.00000E+00 0.00000E+00 0.00000E+00 0.00000E+00 0.00000E+00 0.00000E+00 0.00000E+00 0.00000E+00 0.00000E+00 0.00000E+00 0.00000E+00 0.00000E+00 0.00000E+00 0.00000E+00 0.00000E+00 0.00000E+00 0.00000E+00 0.00000E+00 0.00000E+00 0.00000E+00 0.00000E+00 0.00000E+00 0.00000E+00 0.00000E+00 0.00000E+00 0.00000E+00 0.00000E+00 0.00000E+00 0.00000E+00 0.00000E+00 0.00000E+00 0.00000E+00 0.00000E+00 0.00000E+00 0.00000E+00

0.00000E+00 0.00000E+00 0.00000E+00 0.00000E+00 0.00000E+00 0.00000E+00 0.00000E+00 0.00000E+00 0.00000E+00 0.00000E+00 0.00000E+00 0.00000E+00 0.00000E+00 0.00000E+00 0.00000E+00 0.00000E+00 0.00000E+00 0.00000E+00 0.00000E+00 0.00000E+00 0.00000E+00 0.00000E+00 0.00000E+00 0.00000E+00 0.00000E+00 0.00000E+00 0.00000E+00 0.00000E+00 0.00000E+00 0.00000E+00 0.00000E+00 0.00000E+00 0.00000E+00 0.00000E+00 0.00000E+00 0.00000E+00 0.00000E+00 0.00000E+00 0.00000E+00 0.00000E+00

0.00000E+00 0.00000E+00 0.00000E+00 0.00000E+00 0.00000E+00 0.00000E+00 0.00000E+00 0.00000E+00 0.00000E+00 0.00000E+00 0.00000E+00 0.00000E+00 0.00000E+00 0.00000E+00 0.00000E+00 0.00000E+00 0.00000E+00 0.00000E+00 0.00000E+00 0.00000E+00 0.00000E+00 0.00000E+00 0.00000E+00 0.00000E+00 0.00000E+00 0.00000E+00 0.00000E+00 0.00000E+00 0.00000E+00 0.00000E+00 0.00000E+00 0.00000E+00 0.00000E+00 0.00000E+00 0.00000E+00 0.00000E+00 0.00000E+00 0.00000E+00 0.00000E+00 0.00000E+00

0.00000E+00 0.00000E+00 0.00000E+00 0.00000E+00 0.00000E+00 0.00000E+00 0.00000E+00 0.00000E+00 0.00000E+00 0.00000E+00 0.00000E+00 0.00000E+00 0.00000E+00 0.00000E+00 0.00000E+00 0.00000E+00 0.00000E+00 0.00000E+00 0.00000E+00 0.00000E+00 0.00000E+00 0.00000E+00 0.00000E+00 0.00000E+00 0.00000E+00 0.00000E+00 0.00000E+00 0.00000E+00 0.00000E+00 0.00000E+00 0.00000E+00 0.00000E+00 0.00000E+00 0.00000E+00 0.00000E+00 0.00000E+00 0.00000E+00 0.00000E+00 0.00000E+00 0.00000E+00

0.00000E+00 0.00000E+00 0.00000E+00 0.00000E+00 0.00000E+00 0.00000E+00 0.00000E+00 0.00000E+00 0.00000E+00 0.00000E+00 0.00000E+00 0.00000E+00 0.00000E+00 0.00000E+00 0.00000E+00 0.00000E+00 0.00000E+00 0.00000E+00 0.00000E+00 0.00000E+00 0.00000E+00 0.00000E+00 0.00000E+00 0.00000E+00 0.00000E+00 0.00000E+00 0.00000E+00 0.00000E+00 0.00000E+00 0.00000E+00 0.00000E+00 0.00000E+00 0.00000E+00 0.00000E+00 0.00000E+00 0.00000E+00 0.00000E+00 0.00000E+00 0.00000E+00 0.00000E+00

0.00000E+00 0.00000E+00 0.00000E+00 0.00000E+00 0.00000E+00 0.00000E+00 0.00000E+00 0.00000E+00 0.00000E+00 0.00000E+00 0.00000E+00 0.00000E+00 0.00000E+00 0.00000E+00 0.00000E+00 0.00000E+00 0.00000E+00 0.00000E+00 0.00000E+00 0.00000E+00 0.00000E+00 0.00000E+00 0.00000E+00 0.00000E+00 0.00000E+00 0.00000E+00 0.00000E+00 0.00000E+00 0.00000E+00 0.00000E+00 0.00000E+00 0.00000E+00 0.00000E+00 0.00000E+00 0.00000E+00 0.00000E+00 0.00000E+00 0.00000E+00 0.00000E+00 0.00000E+00

0.00000E+00 0.00000E+00 0.00000E+00 0.00000E+00 0.00000E+00 0.00000E+00 0.00000E+00 0.00000E+00 0.00000E+00 0.00000E+00 0.00000E+00 0.00000E+00 0.00000E+00 0.00000E+00 0.00000E+00 0.00000E+00 0.00000E+00 0.00000E+00 0.00000E+00 0.00000E+00 0.00000E+00 0.00000E+00 0.00000E+00 0.00000E+00 0.00000E+00 0.00000E+00 0.00000E+00 0.00000E+00 0.00000E+00 0.00000E+00 0.00000E+00 0.00000E+00 0.00000E+00 0.00000E+00 0.00000E+00 0.00000E+00 0.00000E+00 0.00000E+00 0.00000E+00 0.00000E+00

0.00000E+00 0.00000E+00 0.00000E+00 0.00000E+00 0.00000E+00 0.00000E+00 0.00000E+00 0.00000E+00 0.00000E+00 0.00000E+00 0.00000E+00 0.00000E+00 0.00000E+00 0.00000E+00 0.00000E+00 0.00000E+00 0.00000E+00 0.00000E+00 0.00000E+00 0.00000E+00 0.00000E+00 0.00000E+00 0.00000E+00 0.00000E+00 0.00000E+00 0.00000E+00 0.00000E+00 0.00000E+00 0.00000E+00 0.00000E+00 0.00000E+00 0.00000E+00 0.00000E+00 0.00000E+00 0.00000E+00 0.00000E+00 0.00000E+00 0.00000E+00 0.00000E+00 0.00000E+00

0.00000E+00 0.00000E+00 0.00000E+00 0.00000E+00 0.00000E+00 0.00000E+00 0.00000E+00 0.00000E+00 0.00000E+00 0.00000E+00 0.00000E+00 0.00000E+00 0.00000E+00 0.00000E+00 0.00000E+00 0.00000E+00 0.00000E+00 0.00000E+00 0.00000E+00 0.00000E+00 0.00000E+00 0.00000E+00 0.00000E+00 0.00000E+00 0.00000E+00 0.00000E+00 0.00000E+00 0.00000E+00 0.00000E+00 0.00000E+00 0.00000E+00 0.00000E+00 0.00000E+00 0.00000E+00 0.00000E+00 0.00000E+00 0.00000E+00 0.00000E+00 0.00000E+00 0.00000E+00

0.00000E+00 0.00000E+00 0.00000E+00 0.00000E+00 0.00000E+00 0.00000E+00 0.00000E+00 0.00000E+00 0.00000E+00 0.00000E+00 0.00000E+00 0.00000E+00 0.00000E+00 0.00000E+00 0.00000E+00 0.00000E+00 0.00000E+00 0.00000E+00 0.00000E+00 0.00000E+00 0.00000E+00 0.00000E+00 0.00000E+00 0.00000E+00 0.00000E+00 0.00000E+00 0.00000E+00 0.00000E+00 0.00000E+00 0.00000E+00 0.00000E+00 0.00000E+00 0.00000E+00 0.00000E+00 0.00000E+00 0.00000E+00 0.00000E+00 0.00000E+00 0.00000E+00 0.00000E+00

0.00000E+00 0.00000E+00 0.00000E+00 0.00000E+00 0.00000E+00 0.00000E+00 0.00000E+00 0.00000E+00 0.00000E+00 0.00000E+00 0.00000E+00 0.00000E+00 0.00000E+00 0.00000E+00 0.00000E+00 0.00000E+00 0.00000E+00 0.00000E+00 0.00000E+00 0.00000E+00 0.00000E+00 0.00000E+00 0.00000E+00 0.00000E+00 0.00000E+00 0.00000E+00 0.00000E+00 0.00000E+00 0.00000E+00 0.00000E+00 0.00000E+00 0.00000E+00 0.00000E+00 0.00000E+00 0.00000E+00 0.00000E+00 0.00000E+00 0.00000E+00 0.00000E+00 0.00000E+00

0.00000E+00 0.00000E+00 0.00000E+00 0.00000E+00 0.00000E+00 0.00000E+00 0.00000E+00 0.00000E+00 0.00000E+00 0.00000E+00 0.00000E+00 0.00000E+00 0.00000E+00 0.00000E+00 0.00000E+00 0.00000E+00 0.00000E+00 0.00000E+00 0.00000E+00 0.00000E+00 0.00000E+00 0.00000E+00 0.00000E+00 0.00000E+00 0.00000E+00 0.00000E+00 0.00000E+00 0.00000E+00 0.00000E+00 0.00000E+00 0.00000E+00 0.00000E+00 0.00000E+00 0.00000E+00 0.00000E+00 0.00000E+00 0.00000E+00 0.00000E+00 0.00000E+00 0.00000E+00

0.00000E+00 0.00000E+00 0.00000E+00 0.00000E+00 0.00000E+00 0.00000E+00 0.00000E+00 0.00000E+00 0.00000E+00 0.00000E+00 0.00000E+00 0.00000E+00 0.00000E+00 0.00000E+00 0.00000E+00 0.00000E+00 0.00000E+00 0.00000E+00 0.00000E+00 0.00000E+00 0.00000E+00 0.00000E+00 0.00000E+00 0.00000E+00 0.00000E+00 0.00000E+00 0.00000E+00 0.00000E+00 0.00000E+00 0.00000E+00 0.00000E+00 0.00000E+00 0.00000E+00 0.00000E+00 0.00000E+00 0.00000E+00 0.00000E+00 0.00000E+00 0.00000E+00 0.00000E+00

0.00000E+00 0.00000E+00 0.00000E+00 0.00000E+00 0.00000E+00 0.00000E+00 0.00000E+00 0.00000E+00 0.00000E+00 0.00000E+00 0.00000E+00 0.00000E+00 0.00000E+00 0.00000E+00 0.00000E+00 0.00000E+00 0.00000E+00 0.00000E+00 0.00000E+00 0.00000E+00 0.00000E+00 0.00000E+00 0.00000E+00 0.00000E+00 0.00000E+00 0.00000E+00 0.00000E+00 0.00000E+00 0.00000E+00 0.00000E+00 0.00000E+00 0.00000E+00 0.00000E+00 0.00000E+00 0.00000E+00 0.00000E+00 0.00000E+00 0.00000E+00 0.00000E+00 0.00000E+00

0.00000E+00 0.00000E+00 0.00000E+00 0.00000E+00 0.00000E+00 0.00000E+00 0.00000E+00 0.00000E+00 0.00000E+00 0.00000E+00 0.00000E+00 0.00000E+00 0.00000E+00 0.00000E+00 0.00000E+00 0.00000E+00 0.00000E+00 0.00000E+00 0.00000E+00 0.00000E+00 0.00000E+00 0.00000E+00 0.00000E+00 0.00000E+00 0.00000E+00 0.00000E+00 0.00000E+00 0.00000E+00 0.00000E+00 0.00000E+00 0.00000E+00 0.00000E+00 0.00000E+00 0.00000E+00 0.00000E+00 0.00000E+00 0.00000E+00 0.00000E+00 0.00000E+00 0.00000E+00

0.00000E+00 0.00000E+00 0.00000E+00 0.00000E+00 0.00000E+00 0.00000E+00 0.00000E+00 0.00000E+00 0.00000E+00 0.00000E+00 0.00000E+00 0.00000E+00 0.00000E+00 0.00000E+00 0.00000E+00 0.00000E+00 0.00000E+00 0.00000E+00 0.00000E+00 0.00000E+00 0.00000E+00 0.00000E+00 0.00000E+00 0.00000E+00 0.00000E+00 0.00000E+00 0.00000E+00 0.00000E+00 0.00000E+00 0.00000E+00 0.00000E+00 0.00000E+00 0.00000E+00 0.00000E+00 0.00000E+00 0.00000E+00 0.00000E+00 0.00000E+00 0.00000E+00 0.00000E+00

0.00000E+00 0.00000E+00 0.00000E+00 0.00000E+00 0.00000E+00 0.00000E+00 0.00000E+00 0.00000E+00 0.00000E+00 0.00000E+00 0.00000E+00 0.00000E+00 0.00000E+00 0.00000E+00 0.00000E+00 0.00000E+00 0.00000E+00 0.00000E+00 0.00000E+00 0.00000E+00 0.00000E+00 0.00000E+00 0.00000E+00 0.00000E+00 0.00000E+00 0.00000E+00 0.00000E+00 0.00000E+00 0.00000E+00 0.00000E+00 0.00000E+00 0.00000E+00 0.00000E+00 0.00000E+00 0.00000E+00 0.00000E+00 0.00000E+00 0.00000E+00 0.00000E+00 0.00000E+00

0.00000E+00 0.00000E+00 0.00000E+00 0.00000E+00 0.00000E+00 0.00000E+00 0.00000E+00 0.00000E+00 0.00000E+00 0.00000E+00 0.00000E+00 0.00000E+00 0.00000E+00 0.00000E+00 0.00000E+00 0.00000E+00 0.00000E+00 0.00000E+00 0.00000E+00 0.00000E+00 0.00000E+00 0.00000E+00 0.00000E+00 0.00000E+00 0.00000E+00 0.00000E+00 0.00000E+00 0.00000E+00 0.00000E+00 0.00000E+00 0.00000E+00 0.00000E+00 0.00000E+00 0.00000E+00 0.00000E+00 0.00000E+00 0.00000E+00 0.00000E+00 0.00000E+00 0.00000E+00

0.00000E+00 0.00000E+00 0.00000E+00 0.00000E+00 0.00000E+00 0.00000E+00 0.00000E+00 0.00000E+00 0.00000E+00 0.00000E+00 0.00000E+00 0.00000E+00 0.00000E+00 0.00000E+00 0.00000E+00 0.00000E+00 0.00000E+00 0.00000E+00 0.00000E+00 0.00000E+00 0.00000E+00 0.00000E+00 0.00000E+00 0.00000E+00 0.00000E+00 0.00000E+00 0.00000E+00 0.00000E+00 0.00000E+00 0.00000E+00 0.00000E+00 0.00000E+00 0.00000E+00 0.00000E+00 0.00000E+00 0.00000E+00 0.00000E+00 0.00000E+00 0.00000E+00 0.00000E+00

0.00000E+00 0.00000E+00 0.00000E+00 0.00000E+00 0.00000E+00 0.00000E+00 0.00000E+00 0.00000E+00 0.00000E+00 0.00000E+00 0.00000E+00 0.00000E+00 0.00000E+00 0.00000E+00 0.00000E+00 0.00000E+00 0.00000E+00 0.00000E+00 0.00000E+00 0.00000E+00 0.00000E+00 0.00000E+00 0.00000E+00 0.00000E+00 0.00000E+00 0.00000E+00 0.00000E+00 0.00000E+00 0.00000E+00 0.00000E+00 0.00000E+00 0.00000E+00 0.00000E+00 0.00000E+00 0.00000E+00 0.00000E+00 0.00000E+00 0.00000E+00 0.00000E+00 0.00000E+00

0.00000E+00 0.00000E+00 0.00000E+00 0.00000E+00 0.00000E+00 0.00000E+00 0.00000E+00 0.00000E+00 0.00000E+00 0.00000E+00 0.00000E+00 0.00000E+00 0.00000E+00 0.00000E+00 0.00000E+00 0.00000E+00 0.00000E+00 0.00000E+00 0.00000E+00 0.00000E+00 0.00000E+00 0.00000E+00 0.00000E+00 0.00000E+00 0.00000E+00 0.00000E+00 0.00000E+00 0.00000E+00 0.00000E+00 0.00000E+00 0.00000E+00 0.00000E+00 0.00000E+00 0.00000E+00 0.00000E+00 0.00000E+00 0.00000E+00 0.00000E+00 0.00000E+00 0.00000E+00

0.00000E+00 0.00000E+00 0.00000E+00 0.00000E+00 0.00000E+00 0.00000E+00 0.00000E+00 0.00000E+00 0.00000E+00 0.00000E+00 0.00000E+00 0.00000E+00 0.00000E+00 0.00000E+00 0.00000E+00 0.00000E+00 0.00000E+00 0.00000E+00 0.00000E+00 0.00000E+00 0.00000E+00 0.00000E+00 0.00000E+00 0.00000E+00 0.00000E+00 0.00000E+00 0.00000E+00 0.00000E+00 0.00000E+00 0.00000E+00 0.00000E+00 0.00000E+00 0.00000E+00 0.00000E+00 0.00000E+00 0.00000E+00 0.00000E+00 0.00000E+00 0.00000E+00 0.00000E+00

0.00000E+00 0.00000E+00 0.00000E+00 0.00000E+00 0.00000E+00 0.00000E+00 0.00000E+00 0.00000E+00 0.00000E+00 0.00000E+00 0.00000E+00 0.00000E+00 0.00000E+00 0.00000E+00 0.00000E+00 0.00000E+00 0.00000E+00 0.00000E+00 0.00000E+00 0.00000E+00 0.00000E+00 0.00000E+00 0.00000E+00 0.00000E+00 0.00000E+00 0.00000E+00 0.00000E+00 0.00000E+00 0.00000E+00 0.00000E+00 0.00000E+00 0.00000E+00 0.00000E+00 0.00000E+00 0.00000E+00 0.00000E+00 0.00000E+00 0.00000E+00 0.00000E+00 0.00000E+00

0.00000E+00 0.00000E+00 0.00000E+00 0.00000E+00 0.00000E+00 0.00000E+00 0.00000E+00 0.00000E+00 0.00000E+00 0.00000E+00 0.00000E+00 0.00000E+00 0.00000E+00 0.00000E+00 0.00000E+00 0.00000E+00 0.00000E+00 0.00000E+00 0.00000E+00 0.00000E+00 0.00000E+00 0.00000E+00 0.00000E+00 0.00000E+00 0.00000E+00 0.00000E+00 0.00000E+00 0.00000E+00 0.00000E+00 0.00000E+00 0.00000E+00 0.00000E+00 0.00000E+00 0.00000E+00 0.00000E+00 0.00000E+00 0.00000E+00 0.00000E+00 0.00000E+00 0.00000E+00

0.00000E+00 0.00000E+00 0.00000E+00 0.00000E+00 0.00000E+00 0.00000E+00 0.00000E+00 0.00000E+00 0.00000E+00 0.00000E+00 0.00000E+00 0.00000E+00 0.00000E+00 0.00000E+00 0.00000E+00 0.00000E+00 0.00000E+00 0.00000E+00 0.00000E+00 0.00000E+00 0.00000E+00 0.00000E+00 0.00000E+00 0.00000E+00 0.00000E+00 0.00000E+00 0.00000E+00 0.00000E+00 0.00000E+00 0.00000E+00 0.00000E+00 0.00000E+00 0.00000E+00 0.00000E+00 0.00000E+00 0.00000E+00 0.00000E+00 0.00000E+00 0.00000E+00 0.00000E+00

0.00000E+00 0.00000E+00 0.00000E+00 0.00000E+00 0.00000E+00 0.00000E+00 0.00000E+00 0.00000E+00 0.00000E+00 0.00000E+00 0.00000E+00 0.00000E+00 0.00000E+00 0.00000E+00 0.00000E+00 0.00000E+00 0.00000E+00 0.00000E+00 0.00000E+00 0.00000E+00 0.00000E+00 0.00000E+00 0.00000E+00 0.00000E+00 0.00000E+00 0.00000E+00 0.00000E+00 0.00000E+00 0.00000E+00 0.00000E+00 0.00000E+00 0.00000E+00 0.00000E+00 0.00000E+00 0.00000E+00 0.00000E+00 0.00000E+00 0.00000E+00 0.00000E+00 0.00000E+00

0.00000E+00 0.00000E+00 0.00000E+00 0.00000E+00 0.00000E+00 0.00000E+00 0.00000E+00 0.00000E+00 0.00000E+00 0.00000E+00 0.00000E+00 0.00000E+00 0.00000E+00 0.00000E+00 0.00000E+00 0.00000E+00 0.00000E+00 0.00000E+00 0.00000E+00 0.00000E+00 0.00000E+00 0.00000E+00 0.00000E+00 0.00000E+00 0.00000E+00 0.00000E+00 0.00000E+00 0.00000E+00 0.00000E+00 0.00000E+00 0.00000E+00 0.00000E+00 0.00000E+00 0.00000E+00 0.00000E+00 0.00000E+00 0.00000E+00 0.00000E+00 0.00000E+00 0.00000E+00

0.00000E+00 0.00000E+00 0.00000E+00 0.00000E+00 0.00000E+00 0.00000E+00 0.00000E+00 0.00000E+00 0.00000E+00 0.00000E+00 0.00000E+00 0.00000E+00 0.00000E+00 0.00000E+00 0.00000E+00 0.00000E+00 0.00000E+00 0.00000E+00 0.00000E+00 0.00000E+00 0.00000E+00 0.00000E+00 0.00000E+00 0.00000E+00 0.00000E+00 0.00000E+00 0.00000E+00 0.00000E+00 0.00000E+00 0.00000E+00 0.00000E+00 0.00000E+00 0.00000E+00 0.00000E+00 0.00000E+00 0.00000E+00 0.00000E+00 0.00000E+00 0.00000E+00 0.00000E+00
