# Supplementary material for: Diamond-inclusion system recording old deep lithosphere conditions at Udachnaya (Siberia)
Source: Sci Rep. 2019 Aug 29;9:12586. doi: 10.1038/s41598-019-48778-x (PMC6715805; doi:10.1038/s41598-019-48778-x)
Supplement: Supplementary file 8 — Supplementary dataset n. 8 [file 41598_2019_48778_MOESM8_ESM.docx]

**Diamond-inclusion system recording old deep lithosphere conditions at Udachnaya (Siberia)**

**Nestola Fabrizio^1^, Zaffiro Gabriele^2^, Mazzucchelli Mattia L.^2^, Nimis Paolo^1^, Andreozzi Giovanni B.^3^, Periotto Benedetta^1^, Princivalle Francesco^4^, Lenaz Davide^4^, Secco Luciano^1^, Pasqualetto Leonardo^1^, Logvinova Alla M.^5,6^, Sobolev Nikolay V.^5,6^, Lorenzetti Alessandra^7^, Harris Jeffrey W.^8^**

***********************************************************************************************************************

* *

* EOSFIT7c *

* *

* Authors: RJ ANGEL, J GONZALEZ-PLATAS, M ALVARO *

* Version date: 19-June-2017 *

* Version: 7.40 *

* *

* *

* INCLUDING LEAST SQUARES BY I.G. WOOD & J. PIPER AND USING THE CrysFML EOS MODULE. *

* *

* EosFit7c: Angel, Gonzalez-Platas, Alvaro (2014) Zeitschrift fuer Kristallographie, 229:405-419. *

* Phase Transitions: Angel et al (2017) Contribs Min Pet, DOI:10.1007/s00410-017-1349-x *

* Fitting Moduli: Milani et al. (2017) Amer Min, DOI:10.2138/am-2017-5855 *

* *

* Development supported by ERC grant 307322, INDIMEDEA to F. Nestola, *

* SIR-MIUR grant RBSI140351, MILE DEEp, to M. Alvaro, and *

* MINECO and EU-FEDER in project MAT2016-75586-C4-4-P to J. Gonzalez-Platas *

* *

* *

* *

* Current date and time: 27-MAR-2019 19:51 *

* *

***********************************************************************************************************************

EosFit parameter file

This is a fixed-format file. If you edit this file, make sure you do not move anything!

It is safer to change the parameters by loading the file to EosFit, and saving the file after making changes

____________________________________________________________________________________________________________

Title = Magnesiochromite (Ac139 sample) BM2-HP EoS to P-V-T data

Savedate = 27-MAR-2019 at 19:51

Comment =Fitted to P-V data of Nestola et al. (2018); Pmax = 7.85 GPa;

Comment =Fitted to T-V data of Singh et al. (1975), Grimes and Al-Ajaj (1992), Wang et al. (2012); Tmax = 1273 K;

Comment =Volume data rescaled to its own V0 and V/V0 = 1 kept fixed during the fit;

Comment =gamma0 and q kept fixed during the fit (not relevant for the Holland-Powell model);

Comment = K' fixed from the EoS model used (BM2);

Comment =All the other EoS parameters were refined;

Comment =EoS obtained by Gabriele Zaffiro (University of Pavia).

Model = 2, (Birch-Murnaghan)

Order = 2

Thermal = 6, (HP Thermal Pres)

Cross = 0, (none)

Trans = 0, (none)

Shear = 0, (none)

Type = Volume

Pref = 0.00000

Tref = 298.00000

Pscale =GPa

Vscale =V/V0

Stoich = 1.00000

Param = 1 1.00000 (V0 , Reference pressure volume: units are V/V0)

Param = 2 183.255 (K0 , Bulk modulus: units are GPa)

Param = 3 4.00000 (Kp , dK/dP: dimensionless)

Param = 4 -0.0212 (Kpp , d2K/dP2: units are inverse GPa)

Param = 5 0.00000

Param = 6 0.00000

Param = 7 0.00000

Param = 8 0.00000

Param = 9 0.00000

Param =10 1.66188 (alph0, Constant of thermal expansion at Tref x10^5 K^-1)

Param =11 683.340 (Th_E , Einstein temperature in K)

Param =12 0.00000

Param =13 0.00000

Param =14 0.00000

Param =15 0.00000

Param =16 0.00000

Param =17 0.00000

Param =18 1.00000 (Gamm0, Gruneisen parameter at Tref,Pref)

Param =19 0.00000 (q , Gruneisen power law in V/V0)

Param =20 0.00000

Param =21 0.00000

Param =22 0.00000

Param =23 0.00000

Param =24 0.00000

Param =25 0.00000

Param =26 0.00000

Param =27 0.00000

Param =28 0.00000

Param =29 0.00000

Param =30 Inf

Param =31 0.00000

Param =32 0.00000

Param =33 0.00000

Param =34 0.00000

Param =35 0.00000

Param =36 0.00000

Param =37 0.00000

Param =38 0.00000

Param =39 0.00000

Param =40 0.00000

Variance-Covariance matrix=

0.00000E+00 0.00000E+00 0.00000E+00 0.00000E+00 0.00000E+00 0.00000E+00 0.00000E+00 0.00000E+00 0.00000E+00 0.00000E+00 0.00000E+00 0.00000E+00 0.00000E+00 0.00000E+00 0.00000E+00 0.00000E+00 0.00000E+00 0.00000E+00 0.00000E+00 0.00000E+00 0.00000E+00 0.00000E+00 0.00000E+00 0.00000E+00 0.00000E+00 0.00000E+00 0.00000E+00 0.00000E+00 0.00000E+00 0.00000E+00 0.00000E+00 0.00000E+00 0.00000E+00 0.00000E+00 0.00000E+00 0.00000E+00 0.00000E+00 0.00000E+00 0.00000E+00 0.00000E+00

0.00000E+00 0.29824E+00 0.00000E+00 0.00000E+00 0.00000E+00 0.00000E+00 0.00000E+00 0.00000E+00 0.00000E+00 0.10472E-07-0.14635E-03 0.00000E+00 0.00000E+00 0.00000E+00 0.00000E+00 0.00000E+00 0.00000E+00 0.00000E+00 0.00000E+00 0.00000E+00 0.00000E+00 0.00000E+00 0.00000E+00 0.00000E+00 0.00000E+00 0.00000E+00 0.00000E+00 0.00000E+00 0.00000E+00 0.00000E+00 0.00000E+00 0.00000E+00 0.00000E+00 0.00000E+00 0.00000E+00 0.00000E+00 0.00000E+00 0.00000E+00 0.00000E+00 0.00000E+00

0.00000E+00 0.00000E+00 0.00000E+00 0.00000E+00 0.00000E+00 0.00000E+00 0.00000E+00 0.00000E+00 0.00000E+00 0.00000E+00 0.00000E+00 0.00000E+00 0.00000E+00 0.00000E+00 0.00000E+00 0.00000E+00 0.00000E+00 0.00000E+00 0.00000E+00 0.00000E+00 0.00000E+00 0.00000E+00 0.00000E+00 0.00000E+00 0.00000E+00 0.00000E+00 0.00000E+00 0.00000E+00 0.00000E+00 0.00000E+00 0.00000E+00 0.00000E+00 0.00000E+00 0.00000E+00 0.00000E+00 0.00000E+00 0.00000E+00 0.00000E+00 0.00000E+00 0.00000E+00

0.00000E+00 0.00000E+00 0.00000E+00 0.00000E+00 0.00000E+00 0.00000E+00 0.00000E+00 0.00000E+00 0.00000E+00 0.00000E+00 0.00000E+00 0.00000E+00 0.00000E+00 0.00000E+00 0.00000E+00 0.00000E+00 0.00000E+00 0.00000E+00 0.00000E+00 0.00000E+00 0.00000E+00 0.00000E+00 0.00000E+00 0.00000E+00 0.00000E+00 0.00000E+00 0.00000E+00 0.00000E+00 0.00000E+00 0.00000E+00 0.00000E+00 0.00000E+00 0.00000E+00 0.00000E+00 0.00000E+00 0.00000E+00 0.00000E+00 0.00000E+00 0.00000E+00 0.00000E+00

0.00000E+00 0.00000E+00 0.00000E+00 0.00000E+00 0.00000E+00 0.00000E+00 0.00000E+00 0.00000E+00 0.00000E+00 0.00000E+00 0.00000E+00 0.00000E+00 0.00000E+00 0.00000E+00 0.00000E+00 0.00000E+00 0.00000E+00 0.00000E+00 0.00000E+00 0.00000E+00 0.00000E+00 0.00000E+00 0.00000E+00 0.00000E+00 0.00000E+00 0.00000E+00 0.00000E+00 0.00000E+00 0.00000E+00 0.00000E+00 0.00000E+00 0.00000E+00 0.00000E+00 0.00000E+00 0.00000E+00 0.00000E+00 0.00000E+00 0.00000E+00 0.00000E+00 0.00000E+00

0.00000E+00 0.00000E+00 0.00000E+00 0.00000E+00 0.00000E+00 0.00000E+00 0.00000E+00 0.00000E+00 0.00000E+00 0.00000E+00 0.00000E+00 0.00000E+00 0.00000E+00 0.00000E+00 0.00000E+00 0.00000E+00 0.00000E+00 0.00000E+00 0.00000E+00 0.00000E+00 0.00000E+00 0.00000E+00 0.00000E+00 0.00000E+00 0.00000E+00 0.00000E+00 0.00000E+00 0.00000E+00 0.00000E+00 0.00000E+00 0.00000E+00 0.00000E+00 0.00000E+00 0.00000E+00 0.00000E+00 0.00000E+00 0.00000E+00 0.00000E+00 0.00000E+00 0.00000E+00

0.00000E+00 0.00000E+00 0.00000E+00 0.00000E+00 0.00000E+00 0.00000E+00 0.00000E+00 0.00000E+00 0.00000E+00 0.00000E+00 0.00000E+00 0.00000E+00 0.00000E+00 0.00000E+00 0.00000E+00 0.00000E+00 0.00000E+00 0.00000E+00 0.00000E+00 0.00000E+00 0.00000E+00 0.00000E+00 0.00000E+00 0.00000E+00 0.00000E+00 0.00000E+00 0.00000E+00 0.00000E+00 0.00000E+00 0.00000E+00 0.00000E+00 0.00000E+00 0.00000E+00 0.00000E+00 0.00000E+00 0.00000E+00 0.00000E+00 0.00000E+00 0.00000E+00 0.00000E+00

0.00000E+00 0.00000E+00 0.00000E+00 0.00000E+00 0.00000E+00 0.00000E+00 0.00000E+00 0.00000E+00 0.00000E+00 0.00000E+00 0.00000E+00 0.00000E+00 0.00000E+00 0.00000E+00 0.00000E+00 0.00000E+00 0.00000E+00 0.00000E+00 0.00000E+00 0.00000E+00 0.00000E+00 0.00000E+00 0.00000E+00 0.00000E+00 0.00000E+00 0.00000E+00 0.00000E+00 0.00000E+00 0.00000E+00 0.00000E+00 0.00000E+00 0.00000E+00 0.00000E+00 0.00000E+00 0.00000E+00 0.00000E+00 0.00000E+00 0.00000E+00 0.00000E+00 0.00000E+00

0.00000E+00 0.00000E+00 0.00000E+00 0.00000E+00 0.00000E+00 0.00000E+00 0.00000E+00 0.00000E+00 0.00000E+00 0.00000E+00 0.00000E+00 0.00000E+00 0.00000E+00 0.00000E+00 0.00000E+00 0.00000E+00 0.00000E+00 0.00000E+00 0.00000E+00 0.00000E+00 0.00000E+00 0.00000E+00 0.00000E+00 0.00000E+00 0.00000E+00 0.00000E+00 0.00000E+00 0.00000E+00 0.00000E+00 0.00000E+00 0.00000E+00 0.00000E+00 0.00000E+00 0.00000E+00 0.00000E+00 0.00000E+00 0.00000E+00 0.00000E+00 0.00000E+00 0.00000E+00

0.00000E+00 0.10472E-07 0.00000E+00 0.00000E+00 0.00000E+00 0.00000E+00 0.00000E+00 0.00000E+00 0.00000E+00 0.51648E-03-0.36246E+00 0.00000E+00 0.00000E+00 0.00000E+00 0.00000E+00 0.00000E+00 0.00000E+00 0.00000E+00 0.00000E+00 0.00000E+00 0.00000E+00 0.00000E+00 0.00000E+00 0.00000E+00 0.00000E+00 0.00000E+00 0.00000E+00 0.00000E+00 0.00000E+00 0.00000E+00 0.00000E+00 0.00000E+00 0.00000E+00 0.00000E+00 0.00000E+00 0.00000E+00 0.00000E+00 0.00000E+00 0.00000E+00 0.00000E+00

0.00000E+00-0.14635E-03 0.00000E+00 0.00000E+00 0.00000E+00 0.00000E+00 0.00000E+00 0.00000E+00 0.00000E+00-0.36246E+00 0.26080E+03 0.00000E+00 0.00000E+00 0.00000E+00 0.00000E+00 0.00000E+00 0.00000E+00 0.00000E+00 0.00000E+00 0.00000E+00 0.00000E+00 0.00000E+00 0.00000E+00 0.00000E+00 0.00000E+00 0.00000E+00 0.00000E+00 0.00000E+00 0.00000E+00 0.00000E+00 0.00000E+00 0.00000E+00 0.00000E+00 0.00000E+00 0.00000E+00 0.00000E+00 0.00000E+00 0.00000E+00 0.00000E+00 0.00000E+00

0.00000E+00 0.00000E+00 0.00000E+00 0.00000E+00 0.00000E+00 0.00000E+00 0.00000E+00 0.00000E+00 0.00000E+00 0.00000E+00 0.00000E+00 0.00000E+00 0.00000E+00 0.00000E+00 0.00000E+00 0.00000E+00 0.00000E+00 0.00000E+00 0.00000E+00 0.00000E+00 0.00000E+00 0.00000E+00 0.00000E+00 0.00000E+00 0.00000E+00 0.00000E+00 0.00000E+00 0.00000E+00 0.00000E+00 0.00000E+00 0.00000E+00 0.00000E+00 0.00000E+00 0.00000E+00 0.00000E+00 0.00000E+00 0.00000E+00 0.00000E+00 0.00000E+00 0.00000E+00

0.00000E+00 0.00000E+00 0.00000E+00 0.00000E+00 0.00000E+00 0.00000E+00 0.00000E+00 0.00000E+00 0.00000E+00 0.00000E+00 0.00000E+00 0.00000E+00 0.00000E+00 0.00000E+00 0.00000E+00 0.00000E+00 0.00000E+00 0.00000E+00 0.00000E+00 0.00000E+00 0.00000E+00 0.00000E+00 0.00000E+00 0.00000E+00 0.00000E+00 0.00000E+00 0.00000E+00 0.00000E+00 0.00000E+00 0.00000E+00 0.00000E+00 0.00000E+00 0.00000E+00 0.00000E+00 0.00000E+00 0.00000E+00 0.00000E+00 0.00000E+00 0.00000E+00 0.00000E+00

0.00000E+00 0.00000E+00 0.00000E+00 0.00000E+00 0.00000E+00 0.00000E+00 0.00000E+00 0.00000E+00 0.00000E+00 0.00000E+00 0.00000E+00 0.00000E+00 0.00000E+00 0.00000E+00 0.00000E+00 0.00000E+00 0.00000E+00 0.00000E+00 0.00000E+00 0.00000E+00 0.00000E+00 0.00000E+00 0.00000E+00 0.00000E+00 0.00000E+00 0.00000E+00 0.00000E+00 0.00000E+00 0.00000E+00 0.00000E+00 0.00000E+00 0.00000E+00 0.00000E+00 0.00000E+00 0.00000E+00 0.00000E+00 0.00000E+00 0.00000E+00 0.00000E+00 0.00000E+00

0.00000E+00 0.00000E+00 0.00000E+00 0.00000E+00 0.00000E+00 0.00000E+00 0.00000E+00 0.00000E+00 0.00000E+00 0.00000E+00 0.00000E+00 0.00000E+00 0.00000E+00 0.00000E+00 0.00000E+00 0.00000E+00 0.00000E+00 0.00000E+00 0.00000E+00 0.00000E+00 0.00000E+00 0.00000E+00 0.00000E+00 0.00000E+00 0.00000E+00 0.00000E+00 0.00000E+00 0.00000E+00 0.00000E+00 0.00000E+00 0.00000E+00 0.00000E+00 0.00000E+00 0.00000E+00 0.00000E+00 0.00000E+00 0.00000E+00 0.00000E+00 0.00000E+00 0.00000E+00

0.00000E+00 0.00000E+00 0.00000E+00 0.00000E+00 0.00000E+00 0.00000E+00 0.00000E+00 0.00000E+00 0.00000E+00 0.00000E+00 0.00000E+00 0.00000E+00 0.00000E+00 0.00000E+00 0.00000E+00 0.00000E+00 0.00000E+00 0.00000E+00 0.00000E+00 0.00000E+00 0.00000E+00 0.00000E+00 0.00000E+00 0.00000E+00 0.00000E+00 0.00000E+00 0.00000E+00 0.00000E+00 0.00000E+00 0.00000E+00 0.00000E+00 0.00000E+00 0.00000E+00 0.00000E+00 0.00000E+00 0.00000E+00 0.00000E+00 0.00000E+00 0.00000E+00 0.00000E+00

0.00000E+00 0.00000E+00 0.00000E+00 0.00000E+00 0.00000E+00 0.00000E+00 0.00000E+00 0.00000E+00 0.00000E+00 0.00000E+00 0.00000E+00 0.00000E+00 0.00000E+00 0.00000E+00 0.00000E+00 0.00000E+00 0.00000E+00 0.00000E+00 0.00000E+00 0.00000E+00 0.00000E+00 0.00000E+00 0.00000E+00 0.00000E+00 0.00000E+00 0.00000E+00 0.00000E+00 0.00000E+00 0.00000E+00 0.00000E+00 0.00000E+00 0.00000E+00 0.00000E+00 0.00000E+00 0.00000E+00 0.00000E+00 0.00000E+00 0.00000E+00 0.00000E+00 0.00000E+00

0.00000E+00 0.00000E+00 0.00000E+00 0.00000E+00 0.00000E+00 0.00000E+00 0.00000E+00 0.00000E+00 0.00000E+00 0.00000E+00 0.00000E+00 0.00000E+00 0.00000E+00 0.00000E+00 0.00000E+00 0.00000E+00 0.00000E+00 0.00000E+00 0.00000E+00 0.00000E+00 0.00000E+00 0.00000E+00 0.00000E+00 0.00000E+00 0.00000E+00 0.00000E+00 0.00000E+00 0.00000E+00 0.00000E+00 0.00000E+00 0.00000E+00 0.00000E+00 0.00000E+00 0.00000E+00 0.00000E+00 0.00000E+00 0.00000E+00 0.00000E+00 0.00000E+00 0.00000E+00

0.00000E+00 0.00000E+00 0.00000E+00 0.00000E+00 0.00000E+00 0.00000E+00 0.00000E+00 0.00000E+00 0.00000E+00 0.00000E+00 0.00000E+00 0.00000E+00 0.00000E+00 0.00000E+00 0.00000E+00 0.00000E+00 0.00000E+00 0.00000E+00 0.00000E+00 0.00000E+00 0.00000E+00 0.00000E+00 0.00000E+00 0.00000E+00 0.00000E+00 0.00000E+00 0.00000E+00 0.00000E+00 0.00000E+00 0.00000E+00 0.00000E+00 0.00000E+00 0.00000E+00 0.00000E+00 0.00000E+00 0.00000E+00 0.00000E+00 0.00000E+00 0.00000E+00 0.00000E+00

0.00000E+00 0.00000E+00 0.00000E+00 0.00000E+00 0.00000E+00 0.00000E+00 0.00000E+00 0.00000E+00 0.00000E+00 0.00000E+00 0.00000E+00 0.00000E+00 0.00000E+00 0.00000E+00 0.00000E+00 0.00000E+00 0.00000E+00 0.00000E+00 0.00000E+00 0.00000E+00 0.00000E+00 0.00000E+00 0.00000E+00 0.00000E+00 0.00000E+00 0.00000E+00 0.00000E+00 0.00000E+00 0.00000E+00 0.00000E+00 0.00000E+00 0.00000E+00 0.00000E+00 0.00000E+00 0.00000E+00 0.00000E+00 0.00000E+00 0.00000E+00 0.00000E+00 0.00000E+00

0.00000E+00 0.00000E+00 0.00000E+00 0.00000E+00 0.00000E+00 0.00000E+00 0.00000E+00 0.00000E+00 0.00000E+00 0.00000E+00 0.00000E+00 0.00000E+00 0.00000E+00 0.00000E+00 0.00000E+00 0.00000E+00 0.00000E+00 0.00000E+00 0.00000E+00 0.00000E+00 0.00000E+00 0.00000E+00 0.00000E+00 0.00000E+00 0.00000E+00 0.00000E+00 0.00000E+00 0.00000E+00 0.00000E+00 0.00000E+00 0.00000E+00 0.00000E+00 0.00000E+00 0.00000E+00 0.00000E+00 0.00000E+00 0.00000E+00 0.00000E+00 0.00000E+00 0.00000E+00

0.00000E+00 0.00000E+00 0.00000E+00 0.00000E+00 0.00000E+00 0.00000E+00 0.00000E+00 0.00000E+00 0.00000E+00 0.00000E+00 0.00000E+00 0.00000E+00 0.00000E+00 0.00000E+00 0.00000E+00 0.00000E+00 0.00000E+00 0.00000E+00 0.00000E+00 0.00000E+00 0.00000E+00 0.00000E+00 0.00000E+00 0.00000E+00 0.00000E+00 0.00000E+00 0.00000E+00 0.00000E+00 0.00000E+00 0.00000E+00 0.00000E+00 0.00000E+00 0.00000E+00 0.00000E+00 0.00000E+00 0.00000E+00 0.00000E+00 0.00000E+00 0.00000E+00 0.00000E+00

0.00000E+00 0.00000E+00 0.00000E+00 0.00000E+00 0.00000E+00 0.00000E+00 0.00000E+00 0.00000E+00 0.00000E+00 0.00000E+00 0.00000E+00 0.00000E+00 0.00000E+00 0.00000E+00 0.00000E+00 0.00000E+00 0.00000E+00 0.00000E+00 0.00000E+00 0.00000E+00 0.00000E+00 0.00000E+00 0.00000E+00 0.00000E+00 0.00000E+00 0.00000E+00 0.00000E+00 0.00000E+00 0.00000E+00 0.00000E+00 0.00000E+00 0.00000E+00 0.00000E+00 0.00000E+00 0.00000E+00 0.00000E+00 0.00000E+00 0.00000E+00 0.00000E+00 0.00000E+00

0.00000E+00 0.00000E+00 0.00000E+00 0.00000E+00 0.00000E+00 0.00000E+00 0.00000E+00 0.00000E+00 0.00000E+00 0.00000E+00 0.00000E+00 0.00000E+00 0.00000E+00 0.00000E+00 0.00000E+00 0.00000E+00 0.00000E+00 0.00000E+00 0.00000E+00 0.00000E+00 0.00000E+00 0.00000E+00 0.00000E+00 0.00000E+00 0.00000E+00 0.00000E+00 0.00000E+00 0.00000E+00 0.00000E+00 0.00000E+00 0.00000E+00 0.00000E+00 0.00000E+00 0.00000E+00 0.00000E+00 0.00000E+00 0.00000E+00 0.00000E+00 0.00000E+00 0.00000E+00

0.00000E+00 0.00000E+00 0.00000E+00 0.00000E+00 0.00000E+00 0.00000E+00 0.00000E+00 0.00000E+00 0.00000E+00 0.00000E+00 0.00000E+00 0.00000E+00 0.00000E+00 0.00000E+00 0.00000E+00 0.00000E+00 0.00000E+00 0.00000E+00 0.00000E+00 0.00000E+00 0.00000E+00 0.00000E+00 0.00000E+00 0.00000E+00 0.00000E+00 0.00000E+00 0.00000E+00 0.00000E+00 0.00000E+00 0.00000E+00 0.00000E+00 0.00000E+00 0.00000E+00 0.00000E+00 0.00000E+00 0.00000E+00 0.00000E+00 0.00000E+00 0.00000E+00 0.00000E+00

0.00000E+00 0.00000E+00 0.00000E+00 0.00000E+00 0.00000E+00 0.00000E+00 0.00000E+00 0.00000E+00 0.00000E+00 0.00000E+00 0.00000E+00 0.00000E+00 0.00000E+00 0.00000E+00 0.00000E+00 0.00000E+00 0.00000E+00 0.00000E+00 0.00000E+00 0.00000E+00 0.00000E+00 0.00000E+00 0.00000E+00 0.00000E+00 0.00000E+00 0.00000E+00 0.00000E+00 0.00000E+00 0.00000E+00 0.00000E+00 0.00000E+00 0.00000E+00 0.00000E+00 0.00000E+00 0.00000E+00 0.00000E+00 0.00000E+00 0.00000E+00 0.00000E+00 0.00000E+00

0.00000E+00 0.00000E+00 0.00000E+00 0.00000E+00 0.00000E+00 0.00000E+00 0.00000E+00 0.00000E+00 0.00000E+00 0.00000E+00 0.00000E+00 0.00000E+00 0.00000E+00 0.00000E+00 0.00000E+00 0.00000E+00 0.00000E+00 0.00000E+00 0.00000E+00 0.00000E+00 0.00000E+00 0.00000E+00 0.00000E+00 0.00000E+00 0.00000E+00 0.00000E+00 0.00000E+00 0.00000E+00 0.00000E+00 0.00000E+00 0.00000E+00 0.00000E+00 0.00000E+00 0.00000E+00 0.00000E+00 0.00000E+00 0.00000E+00 0.00000E+00 0.00000E+00 0.00000E+00

0.00000E+00 0.00000E+00 0.00000E+00 0.00000E+00 0.00000E+00 0.00000E+00 0.00000E+00 0.00000E+00 0.00000E+00 0.00000E+00 0.00000E+00 0.00000E+00 0.00000E+00 0.00000E+00 0.00000E+00 0.00000E+00 0.00000E+00 0.00000E+00 0.00000E+00 0.00000E+00 0.00000E+00 0.00000E+00 0.00000E+00 0.00000E+00 0.00000E+00 0.00000E+00 0.00000E+00 0.00000E+00 0.00000E+00 0.00000E+00 0.00000E+00 0.00000E+00 0.00000E+00 0.00000E+00 0.00000E+00 0.00000E+00 0.00000E+00 0.00000E+00 0.00000E+00 0.00000E+00

0.00000E+00 0.00000E+00 0.00000E+00 0.00000E+00 0.00000E+00 0.00000E+00 0.00000E+00 0.00000E+00 0.00000E+00 0.00000E+00 0.00000E+00 0.00000E+00 0.00000E+00 0.00000E+00 0.00000E+00 0.00000E+00 0.00000E+00 0.00000E+00 0.00000E+00 0.00000E+00 0.00000E+00 0.00000E+00 0.00000E+00 0.00000E+00 0.00000E+00 0.00000E+00 0.00000E+00 0.00000E+00 0.00000E+00 0.00000E+00 0.00000E+00 0.00000E+00 0.00000E+00 0.00000E+00 0.00000E+00 0.00000E+00 0.00000E+00 0.00000E+00 0.00000E+00 0.00000E+00

0.00000E+00 0.00000E+00 0.00000E+00 0.00000E+00 0.00000E+00 0.00000E+00 0.00000E+00 0.00000E+00 0.00000E+00 0.00000E+00 0.00000E+00 0.00000E+00 0.00000E+00 0.00000E+00 0.00000E+00 0.00000E+00 0.00000E+00 0.00000E+00 0.00000E+00 0.00000E+00 0.00000E+00 0.00000E+00 0.00000E+00 0.00000E+00 0.00000E+00 0.00000E+00 0.00000E+00 0.00000E+00 0.00000E+00 0.00000E+00 0.00000E+00 0.00000E+00 0.00000E+00 0.00000E+00 0.00000E+00 0.00000E+00 0.00000E+00 0.00000E+00 0.00000E+00 0.00000E+00

0.00000E+00 0.00000E+00 0.00000E+00 0.00000E+00 0.00000E+00 0.00000E+00 0.00000E+00 0.00000E+00 0.00000E+00 0.00000E+00 0.00000E+00 0.00000E+00 0.00000E+00 0.00000E+00 0.00000E+00 0.00000E+00 0.00000E+00 0.00000E+00 0.00000E+00 0.00000E+00 0.00000E+00 0.00000E+00 0.00000E+00 0.00000E+00 0.00000E+00 0.00000E+00 0.00000E+00 0.00000E+00 0.00000E+00 0.00000E+00 0.00000E+00 0.00000E+00 0.00000E+00 0.00000E+00 0.00000E+00 0.00000E+00 0.00000E+00 0.00000E+00 0.00000E+00 0.00000E+00

0.00000E+00 0.00000E+00 0.00000E+00 0.00000E+00 0.00000E+00 0.00000E+00 0.00000E+00 0.00000E+00 0.00000E+00 0.00000E+00 0.00000E+00 0.00000E+00 0.00000E+00 0.00000E+00 0.00000E+00 0.00000E+00 0.00000E+00 0.00000E+00 0.00000E+00 0.00000E+00 0.00000E+00 0.00000E+00 0.00000E+00 0.00000E+00 0.00000E+00 0.00000E+00 0.00000E+00 0.00000E+00 0.00000E+00 0.00000E+00 0.00000E+00 0.00000E+00 0.00000E+00 0.00000E+00 0.00000E+00 0.00000E+00 0.00000E+00 0.00000E+00 0.00000E+00 0.00000E+00

0.00000E+00 0.00000E+00 0.00000E+00 0.00000E+00 0.00000E+00 0.00000E+00 0.00000E+00 0.00000E+00 0.00000E+00 0.00000E+00 0.00000E+00 0.00000E+00 0.00000E+00 0.00000E+00 0.00000E+00 0.00000E+00 0.00000E+00 0.00000E+00 0.00000E+00 0.00000E+00 0.00000E+00 0.00000E+00 0.00000E+00 0.00000E+00 0.00000E+00 0.00000E+00 0.00000E+00 0.00000E+00 0.00000E+00 0.00000E+00 0.00000E+00 0.00000E+00 0.00000E+00 0.00000E+00 0.00000E+00 0.00000E+00 0.00000E+00 0.00000E+00 0.00000E+00 0.00000E+00

0.00000E+00 0.00000E+00 0.00000E+00 0.00000E+00 0.00000E+00 0.00000E+00 0.00000E+00 0.00000E+00 0.00000E+00 0.00000E+00 0.00000E+00 0.00000E+00 0.00000E+00 0.00000E+00 0.00000E+00 0.00000E+00 0.00000E+00 0.00000E+00 0.00000E+00 0.00000E+00 0.00000E+00 0.00000E+00 0.00000E+00 0.00000E+00 0.00000E+00 0.00000E+00 0.00000E+00 0.00000E+00 0.00000E+00 0.00000E+00 0.00000E+00 0.00000E+00 0.00000E+00 0.00000E+00 0.00000E+00 0.00000E+00 0.00000E+00 0.00000E+00 0.00000E+00 0.00000E+00

0.00000E+00 0.00000E+00 0.00000E+00 0.00000E+00 0.00000E+00 0.00000E+00 0.00000E+00 0.00000E+00 0.00000E+00 0.00000E+00 0.00000E+00 0.00000E+00 0.00000E+00 0.00000E+00 0.00000E+00 0.00000E+00 0.00000E+00 0.00000E+00 0.00000E+00 0.00000E+00 0.00000E+00 0.00000E+00 0.00000E+00 0.00000E+00 0.00000E+00 0.00000E+00 0.00000E+00 0.00000E+00 0.00000E+00 0.00000E+00 0.00000E+00 0.00000E+00 0.00000E+00 0.00000E+00 0.00000E+00 0.00000E+00 0.00000E+00 0.00000E+00 0.00000E+00 0.00000E+00

0.00000E+00 0.00000E+00 0.00000E+00 0.00000E+00 0.00000E+00 0.00000E+00 0.00000E+00 0.00000E+00 0.00000E+00 0.00000E+00 0.00000E+00 0.00000E+00 0.00000E+00 0.00000E+00 0.00000E+00 0.00000E+00 0.00000E+00 0.00000E+00 0.00000E+00 0.00000E+00 0.00000E+00 0.00000E+00 0.00000E+00 0.00000E+00 0.00000E+00 0.00000E+00 0.00000E+00 0.00000E+00 0.00000E+00 0.00000E+00 0.00000E+00 0.00000E+00 0.00000E+00 0.00000E+00 0.00000E+00 0.00000E+00 0.00000E+00 0.00000E+00 0.00000E+00 0.00000E+00

0.00000E+00 0.00000E+00 0.00000E+00 0.00000E+00 0.00000E+00 0.00000E+00 0.00000E+00 0.00000E+00 0.00000E+00 0.00000E+00 0.00000E+00 0.00000E+00 0.00000E+00 0.00000E+00 0.00000E+00 0.00000E+00 0.00000E+00 0.00000E+00 0.00000E+00 0.00000E+00 0.00000E+00 0.00000E+00 0.00000E+00 0.00000E+00 0.00000E+00 0.00000E+00 0.00000E+00 0.00000E+00 0.00000E+00 0.00000E+00 0.00000E+00 0.00000E+00 0.00000E+00 0.00000E+00 0.00000E+00 0.00000E+00 0.00000E+00 0.00000E+00 0.00000E+00 0.00000E+00

0.00000E+00 0.00000E+00 0.00000E+00 0.00000E+00 0.00000E+00 0.00000E+00 0.00000E+00 0.00000E+00 0.00000E+00 0.00000E+00 0.00000E+00 0.00000E+00 0.00000E+00 0.00000E+00 0.00000E+00 0.00000E+00 0.00000E+00 0.00000E+00 0.00000E+00 0.00000E+00 0.00000E+00 0.00000E+00 0.00000E+00 0.00000E+00 0.00000E+00 0.00000E+00 0.00000E+00 0.00000E+00 0.00000E+00 0.00000E+00 0.00000E+00 0.00000E+00 0.00000E+00 0.00000E+00 0.00000E+00 0.00000E+00 0.00000E+00 0.00000E+00 0.00000E+00 0.00000E+00

0.00000E+00 0.00000E+00 0.00000E+00 0.00000E+00 0.00000E+00 0.00000E+00 0.00000E+00 0.00000E+00 0.00000E+00 0.00000E+00 0.00000E+00 0.00000E+00 0.00000E+00 0.00000E+00 0.00000E+00 0.00000E+00 0.00000E+00 0.00000E+00 0.00000E+00 0.00000E+00 0.00000E+00 0.00000E+00 0.00000E+00 0.00000E+00 0.00000E+00 0.00000E+00 0.00000E+00 0.00000E+00 0.00000E+00 0.00000E+00 0.00000E+00 0.00000E+00 0.00000E+00 0.00000E+00 0.00000E+00 0.00000E+00 0.00000E+00 0.00000E+00 0.00000E+00 0.00000E+00

0.00000E+00 0.00000E+00 0.00000E+00 0.00000E+00 0.00000E+00 0.00000E+00 0.00000E+00 0.00000E+00 0.00000E+00 0.00000E+00 0.00000E+00 0.00000E+00 0.00000E+00 0.00000E+00 0.00000E+00 0.00000E+00 0.00000E+00 0.00000E+00 0.00000E+00 0.00000E+00 0.00000E+00 0.00000E+00 0.00000E+00 0.00000E+00 0.00000E+00 0.00000E+00 0.00000E+00 0.00000E+00 0.00000E+00 0.00000E+00 0.00000E+00 0.00000E+00 0.00000E+00 0.00000E+00 0.00000E+00 0.00000E+00 0.00000E+00 0.00000E+00 0.00000E+00 0.00000E+00
