# Supplementary material for: Diamond-inclusion system recording old deep lithosphere conditions at Udachnaya (Siberia)
Source: Sci Rep. 2019 Aug 29;9:12586. doi: 10.1038/s41598-019-48778-x (PMC6715805; doi:10.1038/s41598-019-48778-x)
Supplement: Supplementary file 10 — Supplementary materials [file 41598_2019_48778_MOESM10_ESM.docx]

**Diamond-inclusion system recording old deep lithosphere conditions at Udachnaya (Siberia)**

**Nestola Fabrizio^1^, Zaffiro Gabriele^2^, Mazzucchelli Mattia L.^2^, Nimis Paolo^1^, Andreozzi Giovanni B.^3^, Periotto Benedetta^1^, Princivalle Francesco^4^, Lenaz Davide^4^, Secco Luciano^1^, Pasqualetto Leonardo^1^, Logvinova Alla M.^5,6^, Sobolev Nikolay V.^5,6^, Lorenzetti Alessandra^7^, Harris Jeffrey W.^8^**

^1^Dipartimento di Geoscienze, Università degli Studi di Padova, Via Gradenigo 6, I-35131, Padova, Italy

^2^Dipartimento di Scienze della Terra e dell’Ambiente, Università degli Studi di Pavia, Via Ferrata 1, I-27100, Pavia, Italy

^3^Dipartimento di Scienze della Terra, Sapienza Università di Roma, Piazzale Aldo Moro 5, I-00185, Roma, Italy

^4^Dipartimento di Matematica e Geoscienze, Università degli Studi di Trieste, Via Weiss 8, I-34127, Trieste, Italy

^5^Institute of Geology and Mineralogy, Russian Academy of Sciences Siberian Branch, Novosibirsk, 630090, Russia

^6^Department of Geology and Geophysics, Novosibirsk State University, Pirogova 2, 630090, Novosibirsk, Russia

^7^Dipartimento di Ingegneria Industriale, Università degli Studi di Padova, Via Marzolo 9, I-35131, Padova, Italy

^8^School of Geographical and Earth Sciences, University of Glasgow, Glasgow G12 8QQ, UK

**SUPPLEMENTARY MATERIALS**

**Table 1**. Chemical analyses of the Ac139 and MgCr2 samples studied in this work from five and eight different spots covering different areas of the crystals (for Ac139, SiO_2_ and V_2_O_3_ were below the detection limit).

|  | **Ac139** | |  | **MgCr2** | |
| --- | --- | --- | --- | --- | --- |
| **Ox wt%** | **Average** | **Sigma** |  | **Average** | **Sigma** |
| **MgO** | 12.86 | ±0.83 |  | 12.91 | ±0.10 |
| **Al_2_O_3_** | 5.69 | ±0.14 |  | 6.63 | ±0.05 |
| **TiO_2_** | 0.45 | ±0.02 |  | 0.65 | ±0.04 |
| **Cr_2_O_3_** | 65.77 | ±0.49 |  | 62.84 | ±0.11 |
| **MnO** | 0.30 | ±0.04 |  | 0.06 | ±0.06 |
| **FeO** | 14.33 | ±1.02 |  | 14.74 | ±0.09 |
| **NiO** | 0.07 | ±0.03 |  | 0.14 | ±0.09 |
| **SiO_2_** | - | - |  | 0.13 | ±0.04 |
| **V_2_O_3_** | - | - |  | 0.45 | ±0.06 |
| **Total** | 99.47 | ±0.70 |  | 98.55 | ±0.20 |
|  |  |  |  |  |  |

**Table 2**. Unit-cell edge and unit-cell volume at different pressures for the Ac139 sample studied in this work. In parentheses the experimental uncertainties are reported.

| ***P* (GPa)** | ***a* (Å)** | ***V* (Å^3^)** |
| --- | --- | --- |
| 0.00010(1) | 8.3210(2) | 576.15(4) |
| 0.566(4) | 8.3128(2) | 574.44(5) |
| 0.827(5) | 8.3093(3) | 573.70(6) |
| 1.138(7) | 8.3042(3) | 572.66(7) |
| 1.325(8) | 8.3016(2) | 572.11(5) |
| 2.145(8) | 8.2893(3) | 569.57(5) |
| 2.964(7) | 8.2776(3) | 567.16(7) |
| 3.954(7) | 8.2640(3) | 564.38(7) |
| 4.641(9)* | 8.2543(4) | 562.39(8) |
| 5.680(9) | 8.2404(4) | 559.56(8) |
| 5.999(10)* | 8.2359(5) | 558.64(9) |
| 7.054(10) | 8.2223(5) | 555.88(9) |
| 7.852(11) | 8.2121(7) | 553.81(14) |
| *Data measured during decompression. | | |

| **N. data** | **EoS** | ***K*_RT0_ (GPa)** | ***K*_RT0_*′*** | **α_V0_ (×10^-5^ °C^-1^)** | ***Θ*_E_ (°C)** | ***∂K/∂T* (GPa/°C)** | ***χ*^2^** |
| --- | --- | --- | --- | --- | --- | --- | --- |
|  |  |  |  |  |  |  |  |
| H*P* + H*T* + L*T* (53) | BM2 + HP | 183.3(5) | 4* | 1.66(2) | 410(16) | -0.0212(2) | 1.1 |
|  | | | | | | | |
| Datasets (*T* range = -196 – 1000 °C, *P*_max_ = 7.85 GPa): this study (H*P*); ref. 31 (L*T*); ref. 29 (H*T*); ref. 30 (H*T*). Literature data were rescaled to its own *V*_0._ *Fixed parameter as indicated by the *F*_E_ – *f*_E_ plot. The Dataset used to obtain the EoS for Ac139 and the file of the EoS to be read with the EosFit7c software^10,54,55^ are provided with the manuscript. | | | | | | | |

**Table 3.** Pressure –Volume –Temperature EoS for the Ac139 sample studied in this work.

**Table 4.** FTIR data (six measurements) and corresponding residence temperatures (*T*_res_, in °C) for three different possible diamond ages.

| **FTIR data** | | |  |  | ***T_res_*** | | |
| --- | --- | --- | --- | --- | --- | --- | --- |
| **Spectra** | **N_total_ (ppm)** | **%IaB** |  |  | **2.0 Ga** | **3.1 Ga** | **3.5 Ga** |
| **Ud_1** | 265 | 30 |  | ***T*_res_** | 1138 | 1126 | 1122 |
| **Ud_2** | 283 | 36 |  |  | 1143 | 1131 | 1128 |
| **Ud_3** | 268 | 31 |  |  | 1139 | 1127 | 1123 |
| **Ud_4** | 258 | 34 |  |  | 1144 | 1131 | 1128 |
| **Ud_5** | 271 | 32 |  |  | 1140 | 1127 | 1124 |
| **Ud_6** | 256 | 29 |  |  | 1138 | 1125 | 1122 |
|  |  |  |  |  |  |  |  |
| **Average** | 267(27) | 32(3) |  | **Average** | 1140(33) | 1128(32) | 1125(32) |
|  |  |  |  |  |  |  |  |
| Notes: the uncertainties on N_total_ and %IaB are about 10% (see DiaMap software^56,57^); the uncertainty in *T*_res_ is from the propagation of uncertainties on FTIR data and constants^58,59^. | | | | | | | |

**Table 5.** Pressures of entrapment (*P*_trap_, in GPa) for different residual pressures (*P*_inc_, in GPa) for different entrapment temperatures (*T*_rap_). In bold, we reported *T*_trap_ coincident with the residence temperatures, *T*_res_, for MgCr2 diamond host obtained by FTIR data. These data were used to construct the orange entrapment isomeke shown in Figure 4.

| ***T*_trap_** | ***P*_trap_** | ***P*_trap_** | ***P*_trap_** |
| --- | --- | --- | --- |
|  |  |  |  |
|  | *P*_inc_ = 1.001 | *P*_inc_ = 1.073 | *P*_inc_ = 1.145 |
|  |  |  |  |
| 700 | 4.936 | 5.100 | 5.261 |
| 750 | 5.111 | 5.274 | 5.435 |
| 800 | 5.283 | 5.446 | 5.608 |
| 850 | 5.454 | 5.616 | 5.778 |
| 900 | 5.622 | 5.785 | 5.946 |
| 950 | 5.789 | 5.952 | 6.112 |
| 1000 | 5.955 | 6.117 | 6.278 |
| 1050 | 6.119 | 6.281 | 6.441 |
| 1100 | 6.282 | 6.444 | 6.604 |
| **1125** | **6.364** | **6.525** | **6.685** |
| **1140** | **6.412** | **6.574** | **6.734** |
| 1150 | 6.444 | 6.606 | 6.766 |
| 1200 | 6.606 | 6.767 | 6.927 |
| 1250 | 6.765 | 6.927 | 7.086 |
| 1300 | 6.925 | 7.086 | 7.245 |
| 1350 | 7.083 | 7.244 | 7.403 |
| 1400 | 7.241 | 7.402 | 7.561 |

**References**

54. Angel R.J., Gonzalez-Platas J., Alvaro M., 2014a. EosFit7c and a Fortran module (library) for equation of state calculations. Z. Krist. - Cryst. Mater. 229, 405–419. https://doi.org/10.1515/zkri-2013-1711,

55. Gonzalez-Platas, J., Alvaro, M., Nestola, F. & Angel, R. J. EosFit7-GUI: a new GUI tool for equation of state calculations, analyses, and teaching. *J. Appl. Crystallogr.* **49**, 1377–1382 (2016).
